# Comparison of estimated glomerular filtration rate equations on prediction of mortality, kidney failure and acute kidney injury

**DOI:** 10.1093/ndt/gfaf054

**Published:** 2025-03-11

**Authors:** Denise M J Veltkamp, Maarten B Rookmaaker, Mark C H de Groot, Marianne C Verhaar, Wouter W van Solinge, Saskia Haitjema, Robin W M Vernooij

**Affiliations:** Department of Nephrology and Hypertension, University Medical Center Utrecht, Utrecht University, Utrecht, The Netherlands; Central Diagnostic Laboratory, University Medical Center Utrecht, Utrecht University, Utrecht, The Netherlands; Department of Nephrology and Hypertension, University Medical Center Utrecht, Utrecht University, Utrecht, The Netherlands; Central Diagnostic Laboratory, University Medical Center Utrecht, Utrecht University, Utrecht, The Netherlands; Department of Nephrology and Hypertension, University Medical Center Utrecht, Utrecht University, Utrecht, The Netherlands; Central Diagnostic Laboratory, University Medical Center Utrecht, Utrecht University, Utrecht, The Netherlands; Central Diagnostic Laboratory, University Medical Center Utrecht, Utrecht University, Utrecht, The Netherlands; Department of Nephrology and Hypertension, University Medical Center Utrecht, Utrecht University, Utrecht, The Netherlands; Central Diagnostic Laboratory, University Medical Center Utrecht, Utrecht University, Utrecht, The Netherlands; Julius Centre for Health Sciences and Primary Care, University Medical Center Utrecht, Utrecht University, Utrecht, The Netherlands

**Keywords:** chronic kidney disease, eGFR equations, epidemiology, kidney failure, risk stratification

## Abstract

**Background:**

The Chronic Kidney Disease Epidemiology Collaboration (CKD-EPI)_ASR-NB_2009 estimated glomerular filtration rate (eGFR) equation has shown substantial overestimation of GFR in Europeans, hence new equations have been developed. We examined the effect of introducing the European Kidney Function Consortium (EKFC) or Lund-Malmö revised (LMR) eGFR equations on KDIGO eGFR category classification in a large cohort. We compared the EKFC and LMR equations with the CKD-EPI_ASR-NB_2009 formula in view of discriminative ability of all-cause mortality, kidney failure with replacement therapy (KFRT) and acute kidney injury (AKI) risks across eGFR categories.

**Methods:**

Individuals aged ≥18 years with a serum creatinine measurement (December 2006–July 2024) at University Medical Center Utrecht, were included. Hazard ratios (HRs) were analysed for all outcomes per eGFR category, per equation. Harrell's Concordance index (C-index) was used to assess the ability of risk discrimination across eGFR categories. Whether reclassification between eGFR categories was justified by the occurrence of events, was assessed with net reclassification improvement analysis.

**Results:**

In total, 285 686 individuals were included. Compared with the CKD-EPI_ASR-NB_2009 equation, the EKFC and LMR estimated GFR lower [mean –6.3 (standard deviation, SD 5.3) and –10.7 (SD 6.5) mL/min/1.73^2^, respectively]. The number of individuals with eGFR <60 mL/min/1.73 m^2^ increased 29.0% (EKFC) and 36.4% (LMR). The EKFC predominantly reclassified older individuals, and the LMR older men, to worse eGFR categories. HRs of reclassified individuals to worse eGFR categories were mainly higher compared with the non-reclassified. The EKFC and LMR equations showed equal/improved C-index for mortality (EKFC 0.584/LMR 0.588/CKD-EPI_ASR-NB_2009 0.570), KFRT (0.895/0.900/0.897) and AKI (0.606/0.609/0.599). The LMR equation reclassified more individuals without an event to worse eGFR categories.

**Conclusion:**

eGFR category classification was substantially different when using the EKFC or LMR equation compared with the CKD-EPI_ASR-NB_2009 formula. Both equations showed equal to improved ability of risk stratification across eGFR categories. Shifts in eGFR category classification might significantly impact clinical decisions. Given that we have identified variation between equations, a careful consideration of the advantages and disadvantages of different eGFR equations is essential.

## Graphical Abstract

**Graphical Abstract:**
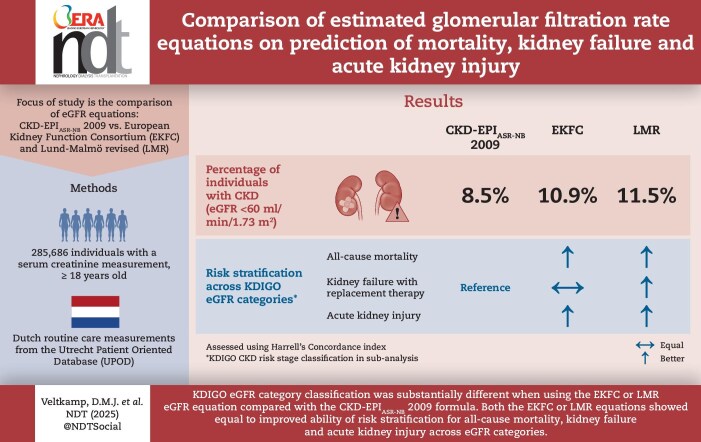

KEY LEARNING POINTS **What was known:**

- The Chronic Kidney Disease Epidemiology Collaboration (CKD-EPI)_ASR-NB_ 2009 equation substantially overestimates glomerular filtration rate (GFR) in White Europeans. The European Kidney Function Consortium (EKFC) and Lund-Malmö revised (LMR) equations are new and both outperform the CKD-EPI_ASR-NB_ 2009 equation in GFR estimation in White Europeans in terms of bias and accuracy [assessed by comparing with measured GFR (mGFR)].

**This study adds:**

- Both the EKFC and LMR equation led mostly to lower estimated GFR (eGFR) compared with the CKD-EPI_ASR-NB_ 2009 equation. This impacted the eGFR category classification. The number of individuals with eGFR <60 mL/min/1.73 m^2^ increased 29.0% and 36.4% using the EKFC and LMR equation, respectively. The EKFC predominantly reclassified older individuals (men and women equally), and the LMR older men, to worse eGFR categories.
- Both the EKFC and LMR equations (especially the LMR equation) showed equal to improved risk stratification across eGFR categories (assessed by Harrell's C-index) compared with the CKD-EPI_ASR-NB_ 2009 equation. The LMR equation reclassified more individuals without an event to worse eGFR categories compared with the EKFC equation.
- In contrast to eGFR category reclassification of the CKD-EPI_AS_ 2021 eGFR equation, the EKFC and LMR equations resulted in significantly fewer incorrect reclassifications of individuals with one of the studied events to better eGFR categories.

**Potential impact:**

- Introduction of the EKFC or LMR equation substantially impacts eGFR category classification (and the KDIGO CKD risk stage classification, as shown in sub-analysis including individuals with measured urinary albumin-to-creatinine ratio).
- Shifts in eGFR category classification might have significant implications for individuals regarding decisions as the initiation of kidney protective measures. Improved targeting of care to individuals most at risk might decrease kidney function decline, even in individuals with early stages of CKD.

## INTRODUCTION

Estimated glomerular filtration rate (eGFR) is commonly measured in routine clinical practice for various purposes, including the identification and monitoring of chronic kidney disease (CKD). eGFR is a proxy for real GFR, which is associated with kidney and cardiovascular outcomes [1]. eGFR, in combination with urinary albumin-to-creatinine ratio (UACR), can be used to categorize individuals in KDIGO CKD risk stages, providing prognostic information on kidney and cardiovascular outcome risks [2].

Most Dutch laboratories use the eGFR Chronic Kidney Disease Epidemiology Collaboration (CKD-EPI) 2009 formula, based on serum creatinine (SCr), age (A), sex (S) and race (R). However, since it is legally prohibited to record race in the Dutch electronic health record (EHR), everyone is assumed to be ‘non-Black’ (NB) (CKD-EPI_ASR-NB_). The CKD-EPI_ASR-NB_ 2009 equation has shown substantial overestimation of GFR in Europeans [3]. Hence, new eGFR equations have been developed, such as the CKD-EPI_AS_ 2021 equation without the race variable, which has been adjusted so that Black and non-Black populations have the same level of bias. Unfortunately, this led to even more overestimation of GFR in Europeans compared with the CKD-EPI_ASR-NB_ 2009 equation [3, 4]. Consequently, it is discouraged to use the CKD-EPI_AS_ 2021 formula in Europe, even though it is recommended for use in the USA [5–7].

Therefore, specifically for the European population, the European Kidney Function Consortium (EKFC) and Lund-Malmö revised (LMR) equations were developed. Both outperform the currently used CKD-EPI_ASR-NB_ 2009 equation for GFR estimation in terms of bias and accuracy in White Europeans [3]. The EKFC and LMR equations both take physiological changes more into account as the EKFC accounts for muscle mass reduction starting at the age of 40 years, while the LMR assumes a non-linear relationship between age and SCr, leading to an accelerated decline in eGFR at older ages [8–10]. Also, the EKFC includes SCr values that are normalized, based on the median SCr value in healthy persons of the same population (Q-value). The use of rescaled SCr values (SCr/Q) compensates for populational differences in SCr levels [10, 11]. The LMR includes a X-coefficient which makes smaller SCr changes less influential on eGFR at higher SCr values using a logaritmic adjustment [9]. In this study, we focused on the KDIGO eGFR category classification in a large Dutch cohort, instead of the accuracy of the GFR estimation. We analysed the ability of the EKFC and LMR equations to discriminate risks of all-cause mortality, kidney failure with kidney replacement therapy (KFRT) and acute kidney injury (AKI) across the different eGFR categories, compared with the CKD-EPI_ASR-NB_ 2009 formula. Sub-analysis across the different KDIGO CKD risk stages were performed in individuals with known UACR.

## MATERIALS AND METHODS

### Study design and inclusion criteria

For this study, SCr values were utilized that were obtained from routine clinical practice in the outpatient departments of University Medical Center Utrecht (UMCU) [12]. Individuals were selected from the Utrecht Patient Oriented Database (UPOD), which is a relational database of routine care data of all individuals that ever visited the UMCU. The structure and content of UPOD have been described in more detail elsewhere [12]. This study was performed in accordance with the declaration of Helsinki and the ethical guidelines of our institution. The institutional review board of the UMCU approved this study (reference 20/672) and waived the need for informed consent as only pseudonymized data were used for this study. Data collection and handling was conducted in accordance with European privacy legislation (General Data Protection Regulation). Individuals aged 18 years or older with a SCr measurement in the UMCU between 30 November 2006 and 27 June 2024, were extracted from UPOD for this study.

### Data cleaning and determination of baseline kidney function

Experienced UPOD data managers followed an ISO 9001-certified multidisciplinary workflow to obtain the data for the study [13]. In brief, this included removing test patients, individuals that opted out for reuse of their data in research, and inclusion of only approved laboratory values according to the laboratory Quality Management System, as well as double pseudonymization before preprocessing by the researcher. The further preprocessing of the data was performed by the researcher. SCr measurements taken prior to 20 March 2013, were adjusted by subtracting 7 µmol/L to align with the calibration level of measurements obtained after this date. The type of SCr analyzers that were used over the years are listed in the supplements (Supplementary data, Table S1). The baseline kidney function was determined based on the first stable SCr measurement in the dataset per individual. SCr measurements taken during clinical admission (and 1 day prior to admission) or while the individual was experiencing AKI, were not used for determining the baseline kidney function in order to minimize the inclusion of instable baseline values. An episode of AKI was defined as the period from the onset of AKI until the SCr level returned to a level that no longer met the KDIGO AKI criteria, with a maximum duration of 3 months, thereby encompassing acute kidney disease that may occur following the AKI.

### Statistical analyses

#### Descriptive statistics

eGFR was expressed through three formulas, using the same SCr measurements: the CKD-EPI_ASR-NB_ 2009 [14], the EKFC [8] and the LMR [9]. For the CKD-EPI_ASR-NB_ 2009 and EKFC formula, all individuals were assumed to be Caucasian. Although it was not the primary aim of this study, we included the results of the comparison of the CKD-EPI_ASR-NB_ 2009 with the CKD-EPI_AS_ 2021 [4] equation in the Supplementary data. The formulas of all eGFR equations, along with a visualization of the differences in calculated eGFR values between the equations, are provided in the supplements [Supplementary data, Table S2 and Fig. S1 (also included as interactive figure)]. Baseline characteristics including SCr, age, sex year of inclusion and follow-up duration are presented as proportions in case of nominal or categorical variables. Continuous variables are presented as mean with standard deviation (SD) for normally distributed variables, and median with interquartile range (IQR) for non-normally distributed variables.

### eGFR assessment and eGFR category classification

First, discrepancies in estimation of GFR were analysed between the CKD-EPI_ASR-NB_ 2009 equation and the other eGFR formulas. Second, to operationalize possible clinical impact, we calculated the proportion of individuals that were reclassified to a different eGFR category when using the EKFC or LMR. The reference eGFR equation for eGFR category classification was the CKD-EPI_ASR-NB_ 2009 equation. eGFR category classification was based on the baseline eGFR per individual, and defined following the eGFR categories according to the KDIGO guideline [2]: eGFR ≥105 (added), eGFR 90–104; eGFR 60–89; eGFR 45–59; eGFR 30–44, eGFR 15–29, eGFR <15 mL/min/1.73 m^2^. Individuals who changed an eGFR category across the different eGFR formulas were defined as ‘reclassified’, and those consistent in one eGFR category as ‘non-reclassified’.

### Risk stratification across eGFR categories

Cox proportional hazards analyses, reporting hazard ratios (HRs) with corresponding 95% confidence intervals (95% CI), were performed to analyse whether reclassified individuals had different risks for all-cause mortality, KFRT and AKI, compared with the non-reclassified individuals. All-cause mortality was either stated in the EHR, otherwise the individual was censored at the last follow-up date. When analysing all-cause mortality, individuals were censored at time of a kidney transplantation. KFRT was defined as initiation of chronic dialysis or kidney transplantation. AKI was defined following the KDIGO criteria [15]. In the analysis of AKI, individuals were censored at kidney replacement therapy initiation. Mortality was treated as a competing risk factor for KFRT and AKI. Detailed definitions of the outcomes are reported in the supplements (Supplementary data, Table S3).

Third, Harrell's Concordance index (C-index) was used to assess whether the eGFR equations accurately categorized individuals into different eGFR categories that effectively discriminate between varying levels of outcome risks over a period of 10 years. Additionally, Net Reclassification Improvement (NRI) over a period of maximum 10 years was assessed to analyse whether the EKFC or LMR provided better predictions than the CKD-EPI_ASR-NB_ 2009 equation by moving individuals to better or worse eGFR categories [16]. NRI is described in more detail in the Supplementary data (Methods). To assess the C-index and NRI, eGFR ≥60 mL/min/1.73 m^2^ was analysed as one eGFR category to align with the KDIGO CKD guidelines.

### Sensitivity analysis

The above analysis were repeated in a subset of individuals with a known UACR. As KDIGO classifies individuals based on their eGFR and UACR into CKD risk categories with their own screening and treatment strategies, we also classified individuals into these risk groups to align with clinical practice (eGFR in mL/min/1.73 m^2^, UACR in mg/mmol): ‘low risk’, eGFR ≥60 and UACR <3; ‘moderate risk’, eGFR 45–59 and UACR <3, or eGFR ≥60 and UACR 3–30; ‘high risk’, eGFR 30–44 and UACR <3, or eGFR 45–59 and UACR 3–30, or eGFR ≥60 and UACR ≥30; ‘very high risk 1’, eGFR 15–29 and UACR <3, or eGFR 15–44 and UACR 3–30, or eGFR 30–59 and UACR ≥30; ‘very high risk 2’, eGFR <15, or eGFR 15–29 and UACR ≥30 [2].

Analyses were performed using R version 4.3.2, and RStudio 2023.09.1 + 494. The R package ‘nephro’ was used to calculate eGFR using the different equations, the ‘nricens’ package to assess the NRI, and ‘plotly’ to generate the interactive supplementary figure [17–19].

## RESULTS

### Characteristics of included individuals

**Figure 1:**
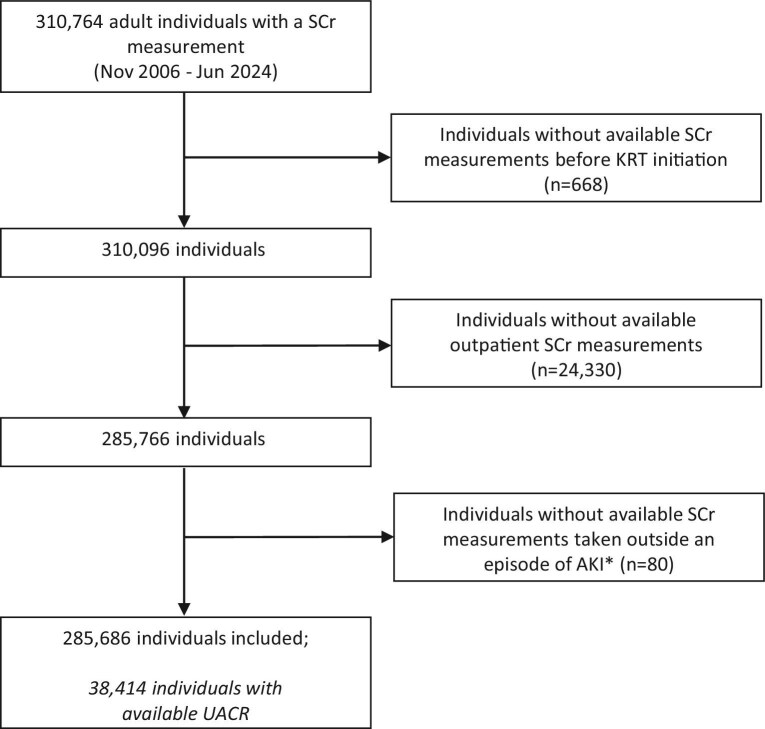
Flow chart for inclusion. KRT, kidney replacement therapy, defined as chronic dialysis or a kidney transplantation. *An episode of AKI was defined as the period from the onset of AKI until the SCr level returned to a level that no longer met the KDIGO AKI criteria, with a maximum duration of 3 months, thereby encompassing acute kidney disease that may occur following the AKI.

In total, 285 686 adult individuals were included in this population-based cohort study (Fig. 1). Mean baseline age was 51.4 (SD 18.6) years, 47.7% were women and the mean baseline SCr concentration was 77.2 (SD 44.7) μmol/L. The mean eGFR with the CKD-EPI_ASR-NB_ 2009 formula was 93.7 (SD 24.2) mL/min/1.73 m^2^. The median follow-up time was 2.0 (IQR 0.3; 6.3) years in individuals with follow-up measurements (Table 1).

**Table 1:** Baseline characteristics and outcomes.

		eGFR category classification (KDIGO) at baseline, based on the CKD-EPI_ASR-NB_ 2009 equation						
	Total	≥105	90–104	60–89	45–59	30–44	15–29	<15
Individuals, *n* (%)	285 686	90 084 (31.5)	79 422 (27.8)	92 018 (32.2)	14 291 (5.0)	6343 (2.2)	2378 (0.8)	1150 (0.4)
Female, *n* (%)	136 272 (47.7)	46 331 (51.4)	35 753 (45.0)	42 969 (46.7)	6718 (47.0)	2962 (46.7)	1094 (46.0)	445 (38.7)
*n* SCr measurements per individual, median (IQR)	2.0 (1.0; 5.0)	2.0 (1.0; 4.0)	2.0 (1.0; 5.0)	2.0 (1.0; 6.0)	2.0 (1.0; 8.0)	3.0 (1.0; 10.0)	3.0 (1.0; 14.0)	2.0 (1.0; 12.0)
FU time in years (excl. no FU), median (IQR)	2.0 (0.3; 6.3)	2.1 (0.3; 6.3)	2.1 (0.4; 6.4)	1.9 (0.3; 6.3)	1.8 (0.3; 5.8)	1.5 (0.4; 4.9)	1.3 (0.4; 4.9)	2.6 (0.8; 7.2)
0 years, *n* (%)	91 864 (32.2)	33 786 (37.5)	26 297 (33.1)	26 877 (29.2)	3224 (22.6)	1181 (18.6)	359 (15.1)	140 (12.2)
>0–1 years, *n* (%)	74 110 (25.9)	21 491 (23.9)	19 675 (24.8)	25 126 (27.3)	4417 (30.9)	2195 (34.6)	894 (37.6)	312 (27.1)
≥1–5 years, *n* (%)	60 491 (21.2)	17 502 (19.4)	16 812 (21.2)	20 021 (21.8)	3485 (24.4)	1701 (26.8)	629 (26.5)	341 (29.7)
≥5–10 years, *n* (%)	33 007 (11.6)	9521 (10.6)	9200 (11.6)	11 174 (12.1)	1864 (13.0)	759 (12.0)	298 (12.5)	191 (16.6)
≥10 years, *n* (%)	26 214 (9.2)	7784 (8.6)	7438 (9.4)	8820 (9.6)	1301 (9.1)	507 (8.0)	198 (8.3)	166 (14.4)
Age, mean (SD)	51.4 (18.6)	33.1 (11.1)	52.5 (13.1)	63.1 (13.9)	71.3 (12.2)	73.0 (13.1)	71.2 (15.2)	60.3 (16.0)
18–30 years, *n* (%)	47 511 (16.6)	39 178 (43.5)	5919 (7.5)	2117 (2.3)	116 (0.8)	79 (1.2)	55 (2.3)	47 (4.1)
30–50 years, *n* (%)	78 519 (27.5)	42 787 (47.5)	21 666 (27.3)	12 739 (13.8)	654 (4.6)	268 (4.2)	175 (7.4)	230 (20.0)
50–65 years, *n* (%)	79 705 (27.9)	7516 (8.3)	37 770 (47.6)	30 044 (32.7)	2651 (18.6)	928 (14.6)	411 (17.3)	385 (33.5)
≥65 years, *n* (%)	79 951 (28.0)	603 (0.7)	14 067 (17.7)	47 118 (51.2)	10 870 (76.1)	5068 (79.9)	1737 (73.0)	488 (42.4)
BMI kg/m^2^, median (IQR)^a^	25.0 (23.0; 28.2)	24.0 (22.0; 28.0)	26.0 (23.0; 29.0)	26.0 (23.0; 29.0)	26.0 (24.0; 29.0)	26.0 (24.0; 29.0)	26.0 (24.0; 30.0)	25.0 (24.0; 29.0)
HbA1c mmol/mol, median (IQR)^b^	37.0 (34.0; 42.0)	35.0 (32.0; 38.0)	37.0 (34.0; 41.2)	39.0 (36.0; 43.0)	41.0 (37.0; 48.0)	41.0 (37.0; 51.0)	40.0 (36.0; 51.0)	35.0 (32.0; 40.0)
LDL mmol/L, median (IQR)^c^	2.9 (2.2; 3.6)	2.8 (2.2; 3.5)	3.0 (2.4; 3.8)	2.9 (2.2; 3.7)	2.6 (1.9; 3.4)	2.5 (1.9; 3.3)	2.4 (1.8; 3.2)	2.3 (1.7; 3.0)
UACR mg/mmol, median (IQR)^d^	0.9 (0.5; 1.9)	0.8 (0.5; 1.6)	0.8 (0.4; 1.6)	0.8 (0.4; 1.9)	1.5 (0.6; 5.1)	3.4 (1.2; 11.6)	8.8 (2.3; 35.1)	25.3 (8.3; 74.7)
SCr µmol/L, mean (SD)	77.2 (44.7)	61.2 (12.8)	69.0 (11.9)	81.2 (13.7)	106.5 (16.5)	138.8 (24.1)	213.9 (50.2)	602.4 (273.4)
eGFRcr mL/min/1.73 m^2^, mean (SD)								
CKD-EPI_ASR-NB_ 2009 equation	93.7 (24.2)	119.3 (12.8)	97.3 (4.3)	78.3 (8.3)	53.4 (4.3)	38.6 (4.2)	23.8 (4.2)	8.8 (3.5)
EKFC equation	87.4 (21.7)	109.8 (9.1)	92.0 (5.5)	73.3 (8.3)	50.0 (4.3)	36.6 (4.0)	23.4 (4.0)	9.4 (3.5)
LMR equation	83.0 (20.3)	103.7 (11.3)	86.8 (4.7)	70.3 (6.9)	49.9 (4.3)	35.1 (4.6)	21.4 (3.2)	10.1 (3.1)
Differences in eGFR between eGFR equations, median (IQR)								
EKFC–CKD-EPI_ASR-NB_ 2009	–6.3 (5.3)	–9.6 (7.1)	–5.3 (3.2)	–5.0 (3.1)	–3.4 (2.0)	–2.0 (1.6)	–0.4 (1.1)	0.7 (0.5)
LMR–CKD-EPI_ASR-NB_ 2009	–10.7 (6.5)	–15.7 (8.3)	–10.5 (2.5)	–8.0 (3.3)	–3.6 (1.7)	–3.5 (1.0)	–2.4 (1.6)	1.4 (0.7)
Year of inclusion^e^, *n* (%)								
December 2006–April 2013	117 276 (41.1)	34 300 (38.1)	31 081 (39.1)	39 859 (43.3)	7013 (49.1)	3216 (50.7)	1248 (52.5)	559 (48.6)
April 2013–October 2019	100 127 (35.0)	31 364 (34.8)	29 142 (36.7)	31 984 (34.8)	4590 (32.1)	1973 (31.1)	724 (30.4)	350 (30.4)
October 2019–July 2024	68 283 (23.9)	24 420 (27.1)	19 199 (24.2)	20 175 (21.9)	2688 (18.8)	1154 (18.2)	406 (17.1)	241 (21.0)
Outcomes								
All-cause mortality, *n* (%)	40 960 (14.3)	4527 (5.0)	10 023 (12.6)	17 202 (18.7)	4763 (33.3)	2737 (43.1)	1230 (51.7)	478 (41.6)
KFRT, *n* (%)	1103 (0.4)	46 (0.1)	36 (0.0)	109 (0.1)	86 (0.6)	127 (2.0)	198 (8.3)	501 (43.6)
AKI (any stage), *n* (%)	9996 (3.5)	1606 (1.8)	2100 (2.6)	3598 (3.9)	1193 (8.3)	789 (12.4)	447 (18.8)	263 (22.9)
AKI stage 2 or 3, *n* (%)	2281 (0.8)	474 (0.5)	561 (0.7)	854 (0.9)	212 (1.5)	95 (1.5)	41 (1.7)	44 (3.8)

Percentage missing: ^a^88.5%, ^b^78.5%, ^c^68.6%, ^d^91.3%.

^e^Based on different SCr analysers.

The mean eGFR calculated with the CKD-EPI_AS_ 2021 equation was 96.9 (SD 23.1) mL/min/1.73 m^2^.

BMI, body mass index; FU, follow-up; HbA1c, glycated hemoglobin; LDL, low-density lipoprotein.

### eGFR category classification

#### EKFC equation

**Figure 2:**
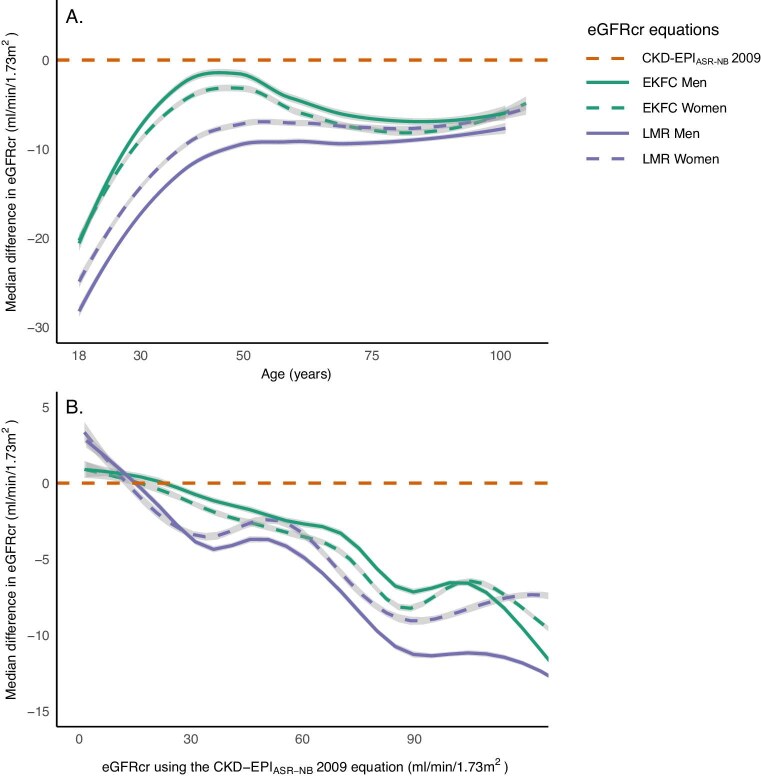
Median differences in eGFRcr between the CKD-EPI_ASR-NB_ 2009 equation as reference, and the EKFC and LMR equations. (**A**) Per age, stratified for sex; (**B**) per eGFRcr as calculated following the CKD-EPI_ASR-NB_ 2009 equation, stratified for sex. The CKD-EPI_ASR-NB_ 2009 equation is represented at y = 0. Values below zero mean that the formula calculates a lower eGFR compared with the CKD-EPI_ASR-NB_ 2009 equation, and vice versa. eGFRcr, eGFR based on SCr.

The mean baseline eGFR following the EKFC formula was 87.4 (SD 21.7) mL/min/1.73 m^2^ (Table 1). The differences between the eGFR calculated with the CKD-EPI_ASR-NB_ 2009 and the EKFC were on average –6.3 (SD 5.3) mL/min/1.73 m^2^. The difference was larger in women [–5.9 (IQR –8.9; –3.4) mL/min/1.73 m^2^] than in men [–4.8 (IQR –7.7; –2.3) mL/min/1.73 m^2^] (Fig. 2A and B, and Supplementary data, Fig. S1). In total, 0.2% (*n* = 526) of all individuals were reclassified to a better eGFR category, and 21.4% (*n* = 61 055) were reclassified to a worse eGFR category. The EKFC classified 7084 individuals (+29.0%) extra as having eGFR <60 mL/min/1.73 m^2^ (Table 2A). Reclassified individuals to worse eGFR categories were mostly older compared with the non-reclassified. The number of reclassified individuals to better eGFR categories was low and included mainly younger individuals (Table 3A).

**Table 2A, B:**
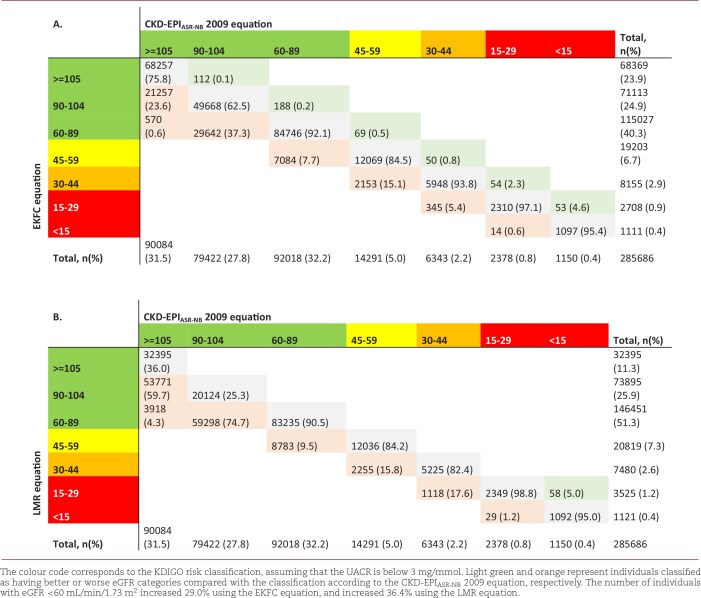
Differences in eGFR category classification between the CKD-EPI_ASR-NB_ 2009, and the (A) EKFC and (B) LMR equation.

**Table 3A, B:**
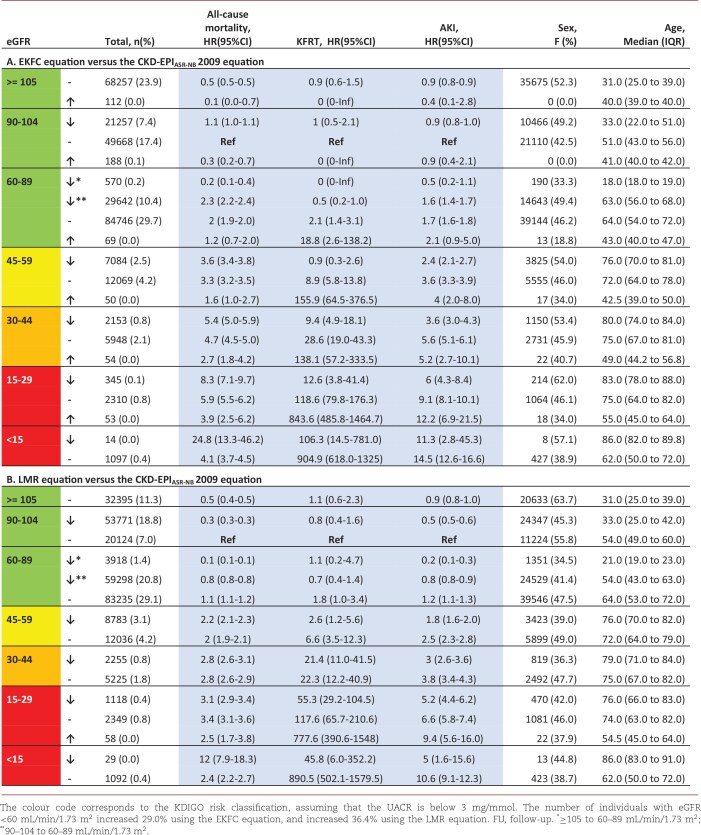
Characteristics and HRs per eGFR category, stratified for reclassified and non-reclassified individuals.

#### LMR equation

The LMR equation led to a mean eGFR of 83.0 (SD 20.3) mL/min/1.73 m^2^ (Table 1). The median difference between the CKD-EPI_ASR-NB_ 2009 and the LMR was –10.7 (SD 6.5) mL/min/1.73 m^2^, which was larger for men [–10.7 (IQR –13.1; –7.7) mL/min/1.73 m^2^] than women [–9.0 (IQR –11.9; –5.5) mL/min/1.73 m^2^] (Fig. 2A and B). In total, 44.2% of all individuals were reclassified to a worse eGFR category (*n* = 127 172). Less than 0.1% (*n* = 58) was reclassified to a better eGFR category. In total, 8783 individuals (+36.4%) were extra classified as having eGFR <60 mL/min/1.73 m^2^ (Table 2B). More men than women were reclassified to worse eGFR categories. These reclassified individuals were slightly older compared with the non-reclassified (Table 3A, B).

### Risk stratification across KDIGO eGFR categories

Supplementary data, Table S5A and B shows if the HRs of reclassified individuals (by the EKFC and the LMR, compared with the classification by the CKD-EPI_ASR-NR_ 2009) are different from their original eGFR category and their new eGFR category. Although reclassified individuals to worse eGFR categories generally had higher outcome risks than those in their original eGFR category, they typically had lower outcome risks compared with others in their new eGFR category, and vice versa. To assess the overall discriminative ability of risks across eGFR categories per eGFR equation, the Harrell's C-index and NRI were analysed.

### EKFC equation

Using the EKFC, risk stratification across the eGFR categories (C-index) was equal to improved compared with the CKD-EPI_ASR-NB_ 2009 equation for mortality (0.584 versus 0.570), AKI (0.551 versus 0.548) and KFRT (0.895 versus 0.897) (Supplementary data, Table S6). The NRI+ for all-cause mortality and AKI were positive, meaning that the majority of reclassified individuals with the outcome were reclassified correctly to worse eGFR categories. However, the NRI+ for KFRT was negative [overall NRI –3.4% (–1.4% (NRI+)/–2.0% (NRI–)]. The negative NRI+ indicates that a larger proportion of reclassified individuals with this outcome were incorrectly reclassified to better eGFR categories, instead to worse eGFR categories. In contrast to the CKD-EPI_AS_ 2021 equation, the EKFC equation resulted in significantly fewer incorrect reclassifications of individuals with one of the events to better eGFR categories (Supplementary data, Table S7).

### LMR equation

The C-index using the LMR instead of the CKD-EPI_ASR-NR_ 2009 showed improved risk stratification across eGFR categories for all outcomes (Supplementary data, Table S6). The overall NRI was positive for all outcomes. The LMR equation reclassified more individuals without an event to worse eGFR categories compared with the EKFC equation. The net proportion of correctly reclassified individuals with an event to worse eGFR categories (NRI+) was positive for all outcomes (this was negative for all outcomes when using the CKD-EPI_AS_ 2021 equation) (Supplementary data, Table S7).

### Sensitivity analysis of risk stratification across KDIGO CKD risk stages, in individuals with available UACR

In total, 599 individuals (+5.3%) were classified as having moderate to very high risk of CKD progression or cardiovascular complications when using the EKFC equation, and 766 individuals (+6.7%) when using the LMR equation. The discrimination of the EKFC and LMR equations were comparable to the CKD-EPI_ASR-NR_ 2009 equation. However the NRI was positive, especially when using the LMR equation (Supplementary data, Tables S8–S11).

## DISCUSSION

We showed large differences in eGFR category classification between the SCr-based EKFC and LMR equations compared with the currently used CKD-EPI_ASR-NB_ 2009 formula. Introducing a new way to calculate eGFR would thus have substantial effect, as it would have increased the number of individuals classified as having eGFR <60 mL/min/1.73 m^2^ with 28.7% and 35.7% at their first presentation, respectively for the EKFC and LMR equations. Mostly older individuals were reclassified to worse eGFR categories by both the EKFC and the LMR equation. The LMR reclassified on average more men to worse categories. Both the EKFC and LMR equations showed equal to improved risk stratification across eGFR categories (assessed by Harrell's C-index) compared with the CKD-EPI_ASR-NB_ 2009 equation. The LMR equation reclassified more individuals without an event to worse eGFR categories. Even though an average change in eGFR of about 5–10 mL/min/1.73 m^2^ may be considered marginal, the shifts in eGFR categories can have significant implications for clinical decision-making in individuals regarding the initiation of kidney protective measures, which is known to delay or even halt kidney function declines [20].

Our findings are in line with results of other studies in large routine care cohorts that compared the CKD-EPI_ASR-NB_ 2009 equation with other eGFR formulas as the CKD-EPI_AS_ 2021 equation. Recent Danish and Swedish studies showed that the perceived prevalence of CKD (defined as an eGFR <60 mL/min/1.73 m^2^) decreased by approximately 25% when using the CKD-EPI_AS_ 2021 instead of the CKD-EPI_ASR-NB_ 2009 formula, which is comparable to the effect it would have in our population (–19.1%, Supplementary data, Table S4) [21, 22]. This can be explained by the overestimation of GFR by the CKD-EPI_AS_ 2021 equation, predominantly in White populations [3]. Importantly, to the best of our knowledge, our study is the first to examine the effect of introducing the EKFC and LMR equation on KDIGO eGFR category (and KDIGO CKD risk stage) classification and their discriminative ability of risks across the categories in a large European routine care dataset. In contrast to the CKD-EPI_AS_ 2021 equation, the EKFC and LMR equations resulted in significantly fewer incorrect reclassifications of individuals with one of the studied events to better eGFR categories.

Age and sex differences in reclassification between the eGFR equations reflect their underlying assumptions. Both equations reclassified older individuals to worse eGFR categories (or CKD stages), possibly due to the age-related adjustments whereby the EKFC specifically assumed muscle mass decrease with ageing from the age of 40 years, while the LMR models steeper decline in eGFR with age. The LMR reclassified more men, which is likely due to the higher SCr threshold (180 µmol/L in men and 150 µmol/L in women) from which SCr increases lead to less steep eGFR reduction (Supplementary data, Fig. S1) [8, 9].

The strength of this study lies in its focus on the prognostic implications of eGFR category (and CKD risk stage in sub-analysis) classification using various eGFR equations, emphasizing their role not only in GFR estimation but also in clinical decision-making. Additionally, information on SCr measurements, including assay type and laboratory methodology, was available and provided, ensuring consistent and comparable eGFR calculations across the study population. The present analysis has several limitations, including that information on the race of included individuals was not available as in Dutch hospitals the race parameter cannot be recorded in the EHR due to the regulatory framework. Nevertheless, the added value of race in eGFR equations is doubted, as race is not necessarily related to SCr level as the biological and social heterogeneity within one racial group can be enormous [23]. A dichotomous factor for race limits clinical applicability to individuals with mixed ethnicity. Besides, the number of individuals of sub-Saharan African descent is relatively low in the Dutch population. Approximately 4.3% has an African migration background, the majority of whom are of North-African descent. About 2.0% of the population has Afro-American backgrounds [24]. Future research in other countries might help to validate the equations and their prognostic implications in other populations [7, 25]. Further, SCr measurements can be variable [26]. To minimize the effect of variable SCr on the prognostic analyses, we excluded measurements that were taken during clinical admission, in the emergency department prior to admission or while the individual was experiencing AKI, for determining the baseline eGFR. SCr values might be higher during pregnancy [27]. We were unable to identify these measurements since information on pregnancy was not available. Due to the retrospective nature of the study and the use of routine care data, some outcomes might have been missed. However, this limitation likely affected the reclassified and non-reclassified individuals equally. The NRI may be less reliable due to the follow-up period being too short to capture the outcome, particularly for KFRT. To mitigate the impact of individuals with short follow-up, we applied a correction in the analysis based on follow-up duration. Additionally, the SCr measurements included in this study were all taken in a tertiary hospital, which may limit the generalizability to the average Dutch population. Validation studies in different populations are needed to confirm whether the observed patterns of reclassification and their clinical implications hold true outside the Dutch context. Though, as previously mentioned, when comparing the observed patterns of reclassification in other European countries, our findings are consistent with these results [20, 21]. This suggests that the reclassification trends observed in this study may be generalizable to other European settings, supporting the broader applicability of the findings. Not every individual included in our study had measurements in urine samples. As a consequence, UACR could only be calculated in a subgroup. Due to bias by indication this may have resulted in a relatively higher proportion of individuals with impaired kidney function in the subgroup with available UACR measurements. Finally, the number of individuals with both SCr and cystatin C measurements was very limited which made reliable analyses on the comparison of cystatin C-based equations impossible.

## CONCLUSION

The eGFR category classification was substantially different when using the EKFC or LMR equation instead of the CKD-EPI_ASR-NB_ 2009 formula. The EKFC and LMR equations showed improved ability of risk stratification across eGFR categories for all-cause mortality, kidney failure with replacement therapy (LMR only) and acute kidney injury compared with the CKD-EPI_ASR-NB_ 2009 equation. Shifts in eGFR category classification might significantly impact clinical decisions. Given that we have identified variation between equations, a careful consideration of the advantages and disadvantages of different eGFR equations is essential.

## Supplementary Material

gfaf054_Supplemental_Files

## Data Availability

The data will be shared on reasonable request to the corresponding author. For inquiries, please contact d.m.j.veltkamp@umcutrecht.nl.
