# Supplementary material for: Comparison of estimated glomerular filtration rate equations on prediction of mortality, kidney failure and acute kidney injury
Source: Nephrol Dial Transplant. 2025 Mar 11;40(10):1876–86. doi: 10.1093/ndt/gfaf054 (PMC12477466; doi:10.1093/ndt/gfaf054)
Supplement: gfaf054_Supplemental_Files [file gfaf054_supplemental_files.zip › Revised_Supplemental_material_10022025.docx]

**Supplemental material**

**Supplemental Table 1.** Serum Creatinine Analysers

**Supplemental Table 2.** eGFR equations
**Supplemental Figure 1.** 3D Scatter plots comparing eGFRcr calculated using the CKD-EPIASR-NB 2009, EKFC and LMR equations, stratified by gender. All age and SCr combinations included (not including data from our cohort).
**Supplemental Table 3.** Definitions of outcomes or included variables
**Supplemental Methods.** Net Reclassification Improvement (NRI)
**Supplemental Table 4.** Differences in eGFR category classification between the CKD-EPI_ASR-NB_ 2009, and the CKD-EPI_AS_ 2021 equation
**Supplemental Table 5A.** Hazard ratios in reclassified individuals, compared to non-reclassified individuals in the original eGFR category
**Supplemental Table 5B.** Hazard ratios in reclassified individuals, compared to non-reclassified individuals in the new eGFR category
**Supplemental Table 6.** Discrimination of eGFR category classification by the different eGFR equations for 10-year outcomes
**Supplemental Table 7.** Net Reclassification Improvement of the reclassification between all eGFR categories over a follow-up period of maximum 10 years, compared to the classification by the CKD-EPI_ASR-NB_ 2009 equation

*Sub-analysis in individuals with a measured urinary albumin-creatinine ratio*

**Supplemental Table 8.** Characteristics of individuals with a measured urinary albumin-creatinine ratio
**Supplemental Table 9.** Differences in KDIGO CKD risk category classification between the CKD-EPI_ASR-NB_ 2009, and the A) CKD-EPI_AS_ 2021 equation, B) EKFC equation, C) LMR equation
**Supplemental Table 10.** Discrimination of KDIGO CKD risk category by the different eGFR equations for 10-year outcomes **Supplemental Table 11.** Net Reclassification Improvement of the reclassification between all KDIGO CKD risk categories over a follow-up period of 10 years, compared to the classification by the CKD-EPI_ASR-NB_ 2009 equation

**References**

**Supplemental table 1.** Serum Creatinine Analysers

| **Period** | **Methodology** | **IDMS calibrated** | **Analysers** | **Manufacturer** |
| --- | --- | --- | --- | --- |
| November 30, 2006 - May 6, 2012 | Kinetic IDMS | 7 µmol/L above calibration level | DxC800 Chemistry analyser | Beckman Coulter, Brea, California |
| May 7, 2012 -  March 19, 2013 | Enzymatic IDMS | 7 µmol/L above calibration level | Au5811 Chemistry analyser | Beckman Coulter, Brea, California |
| March 20, 2013 - September 27, 2019 | Enzymatic IDMS | Yes | Au5811 Chemistry analyser | Beckman Coulter, Brea, California |
| September 28, 2019 - present | Enzymatic IDMS | Yes | Atellica Chemistry analyser | Siemens Healthcare Diagnostics Inc., Tarrytown, USA |

The measurements before March 20, 2013, were retrospectively corrected with 7 µmol/L, as this was the correction factor for isotope dilution mass spectrometry (IDMS) calibration (calibration of the enzymatic SCr assay is traceable to the IDMS reference method as IDMS is considered the gold standard for measuring SCr).

**Supplemental table 2.** eGFR equations

| CKD-EPI_ASR-NB_ 2009 equation^1^ | Males:  If SCr /0.90 ≥ 1: eGFR = 141 x(SCr/0.90)^-1.209^ x 0.9929^Age^ (x 1.159 *if Black individual)*  If SCr /0.90 < 1: eGFR = 141 x(SCr/0.90)^-0.411^ x 0.9929^Age^ (x 1.159 *if Black individual)*  Females:  If SCr /0.70 ≥ 1: eGFR = 144 x(SCr /0.70)^-1.209^ x 0.9929^Age^ (x 1.159 *if Black individual)*  If SCr /0.70 < 1: eGFR = 144 x(SCr /0.70)^-0.329^ x 0.9929^Age^ (x 1.159 *if Black individual)*  *SCr in mg/dL; all individuals were assumed to be Caucasian* |
| --- | --- |
| EKFC equation^2^ | <40 years and SCr/Q<1 : 107.3 x(SCr/Q)^-0.322^  <40 years and SCr/Q≥1 : 107.3 x(SCr/Q)^-1.132^  >40 years and SCr/Q<1: 107.3 x(SCr/Q)^-0.322^ x 0.990^(age-40)^  >40 years and SCr/Q≥1: 107.3 x(SCr/Q)^-1.132^ x 0.990^(age-40)^  Q-value:  18-25 years:  Males: ln(Q)=3.2 + 0.259 x age – 0.543 x ln(age) – 0.00763 x age² + 0.000079 x age³  Females: ln(Q)=3.08 + 0.177 x age – 0.223 x ln(age) – 0.00596 x age² + 0.0000686 x age³  ≥ 25 years:  Caucasian European Males: Q=0.90 mg/dL; Caucasian European Females: Q=0.70 mg/dL  European Black females^3^: Q = Q + 0.04; European Black males^3^: Q = Q + 0.12  *SCr in mg/dL; all individuals were assumed to be Caucasian* |
| LMR equation^4^ | eGFR = 𝑒 ^𝑋−0.0158 × age +0.438 × ln(age)^  Female SCr< 150 μmol/L: X = 2.50 + 0.0121 ×(150 - SCr)  Female SCr≥ 150 μmol/L: X = 2.50 - 0.926 × ln(SCr/ 150)  Male SCr < 180 μmol/L: X = 2.56 + 0.00968 ×(180 - SCr)  Male SCr ≥ 180 μmol/L: X = 2.56 - 0.926 × ln(SCr / 180)  *SCr in μmol/L* |
| CKD-EPI_AS_ 2021 equation^5^ | Males: If SCr≤0.9 mg/dL: 142 x (SCr/0.9)^-0.302^ x 0.9938^age^  Males: If SCr>0.9 mg/dL: 142 x (SCr/0.9)^-1.200^ x 0.9938^age^  Females: SCr≤0.7 mg/dL: 143 x(SCr/0.7)^-0.241^ x 0.9938^age^  Females: SCr>0.7 mg/dL: 143 x (SCr/0.7)^-1.200^ x 0.9938^age^  *SCr in mg/dL* |

CKD-EPI, Chronic Kidney Disease Epidemiology; EKFC, European Kidney Function Consortium; LMR, Lund-Malmö revised ; SCr, serum creatinine. All of the above formulas are applicable for patients of ≥18 years old. For children, use adjusted formulas.

**Supplemental Figure 1.** 3D Scatter plots comparing eGFRcr calculated using the CKD-EPI_ASR-NB_ 2009, EKFC and LMR equations, stratified by gender. All age and SCr combinations included (not including data from our cohort).


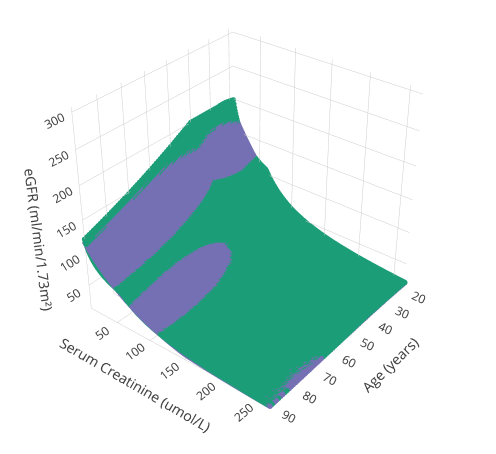

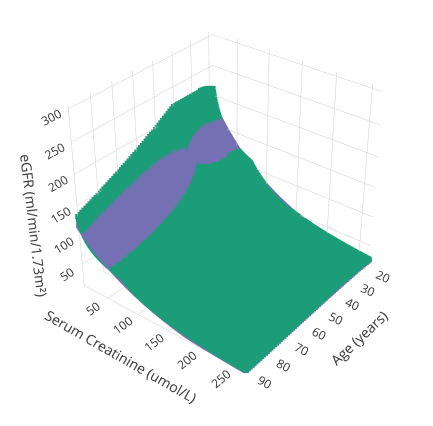

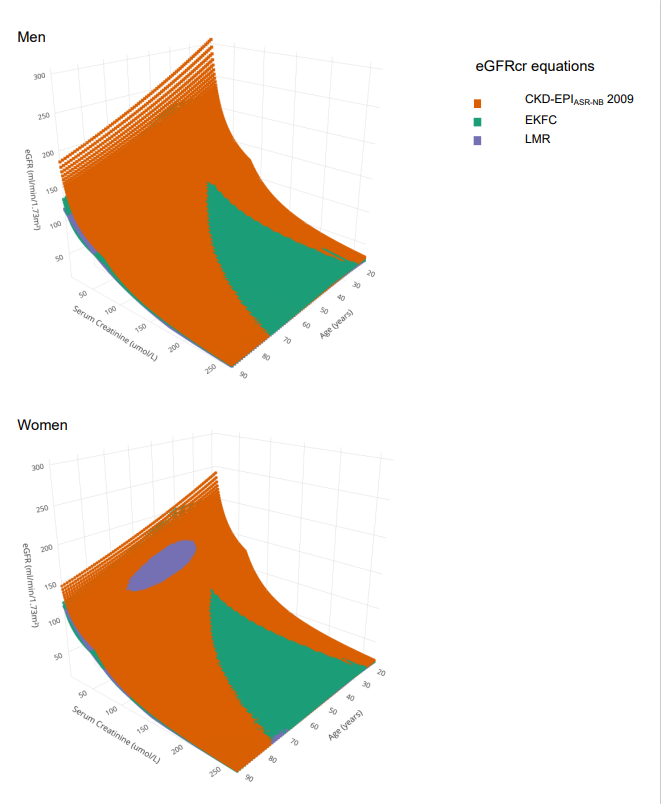


CKD-EPI, Chronic Kidney Disease Epidemiology Collaboration; eGFRcr, estimated glomerular filtration rate using serum creatinine; EKFC, European Kidney Function Consortium; LMR, Lund-Malmö revised

(Interactive figure available)

**Supplemental table 3.** Definitions of outcomes or included variables

| **Outcomes** | |
| --- | --- |
| Acute kidney injury | AKI stage 1: SCr increase of ≥26 umol/L within 48 hours or 1.5x baseline SCr within 7 days;  AKI stage 2: SCr increase of 2-3x baseline SCr within 7 days;  AKI stage 3: SCr increase of ≥3x baseline SCr within 7 days.^6^  If more than one SCr measurement was performed in the specified timeframe, the lowest measured SCr was utilized as the baseline SCr.^7^ |
| All-cause mortality | We could state that the individual had died if this was recorded in the UPOD. Other individuals were censored at the last follow-up date. |
| Kidney failure with kidney replacement therapy | Initiation of chronic dialysis or kidney transplantation |
| **Patient characteristics** | |
| LDL mmol/L | Maximum 365 days around the SCr measurement |
| HbA1c % | Maximum 365 days around the SCr measurement |
| HbA1c mmol/mol | Maximum 365 days around the SCr measurement |
| UACR mg/mmol | Maximum 365 days around the SCr measurement |
| BMI | Length measured from the age of 18 or beyond was used without time restriction. Weight was accepted if it was in a range of 3650 days around the SCr measurement. |

AKI, acute kidney injury; BMI, Body mass index; eGFR, estimated glomerular filtration rate; HbA1c, Glycated hemoglobin; SCr, serum creatinine; UACR, Urinary albumine-to-creatinine Ratio; UPOD, Utrecht Patient Orientated Database

**Supplemental Methods.** Net Reclassification Improvement (NRI)

Net Reclassification Improvement (NRI) is the sum of the net proportion of correctly reclassified individuals with the event (*% of individuals with the event that were correctly reclassified to worse categories* ***-*** *% of individuals with the event that were incorrectly reclassified to better categories*) and the net proportion of correctly reclassified individuals without the event (*% of individuals without the event that were correctly reclassified to better categories* ***-*** *% of individuals without the event that were incorrectly reclassified to worse categories*).^8^ Inverse probability weighting (IPW) was applied to correct for censored data. This gave individuals who were censored earlier a lower weight in the analysis. Positive NRI indicates that the new eGFR equation improves reclassification accuracy, meaning more individuals are correctly categorized into appropriate risk levels. Negative NRI indicates that the new eGFR equation is worse at reclassification compared with the CKD-EPI_ASR-NB_ 2009 equation, meaning it results in more incorrect reclassifications.

**Supplemental Table 4.** Differences in eGFR category classification between the CKD-EPI_ASR-NB_ 2009, and the CKD-EPI_AS_ 2021 equation

A)

|  |  | **CKD-EPI_ASR-NB_ 2009 equation** | | | | | | |  |
| --- | --- | --- | --- | --- | --- | --- | --- | --- | --- |
|  |  | **>=105** | **90-104** | **60-89** | **45-59** | **30-44** | **15-29** | **<15** | **Total, n(%)** |
|  | **>=105** | 90084 (100.0) | 17533 (22.1) |  |  |  |  |  | 107617 (37.7) |
| **CKD-EPI_AS_ 2021 equation** | **90-104** |  | 61889 (77.9) | 22976 (25.0) |  |  |  |  | 84865 (29.7) |
|  | **60-89** |  |  | 69042 (75.0) | 4617 (32.3) |  |  |  | 73659 (25.8) |
|  | **45-59** |  |  |  | 9674 (67.7) | 1678 (26.5) |  |  | 11352 (4.0) |
|  | **30-44** |  |  |  |  | 4665 (73.5) | 480 (20.2) |  | 5145 (1.8) |
|  | **15-29** |  |  |  |  |  | 1898 (79.8) | 96 (8.3) | 1994 (0.7) |
|  | **<15** |  |  |  |  |  |  | 1054 (91.7) | 1054 (0.4) |
|  | **Total, n(%)** | 90084 (31.5) | 79422 (27.8) | 92018 (32.2) | 14291 (5.0) | 6343 (2.2) | 2378 (0.8) | 1150 (0.4) | 285686 |

The color code corresponds to the KDIGO risk classification, assuming that the urinary albumin-to-creatinine ratio is below 3 mg/mmol. Light green and orange represent individuals classified as having better or worse CKD stage compared to the classification according to the CKD-EPI_ASR-NB_ 2009 equation, respectively. The number of individuals with CKD stage 3a-5 decreased 19.1%.

CKD, chronic kidney disease; CKD-EPI, Chronic Kidney Disease Epidemiology Collaboration;

**Supplemental Table 5A.** Hazard ratios in reclassified individuals, compared to non-reclassified individuals in the original eGFR category

| **Non-reclassified** | | | **Reclassified** | | | **Outcomes** |  |  |  |  |  | **non-reclassified/reclassified** | |
| --- | --- | --- | --- | --- | --- | --- | --- | --- | --- | --- | --- | --- | --- |
| **eGFR*** | **n** | **n All-cause mortality/KF with KRT/KF with KRT/AKI** | **eGFR*** | **n** | **n All-cause mortality/KF with KRT/KF with KRT/AKI** | **All-cause mortality, HR(95%CI)** | **All-cause mortality, HR(95%CI)**** | **KF with KRT, HR(95%CI)** | **KF with KRT, HR(95%CI)**** | **AKI, HR(95%CI)** | **AKI, HR(95%CI)**** | **Age, mean(SD)** | **Gender, F %** |
| **Reclassification to worse eGFR categories**  ***EKFC*** | | | | | | *HRs of reclassified individuals versus the HRs in their original* ***better*** *eGFR categories* | | | | | |  |  |
| >= 105 | 68257 | 2698/34/1201 | To 60-89 | 570 | 9/0/6 | 0.4 (0.2-0.8) | 1.6 (0.9-3.2) | 0.0 (0.0-Inf) | 0.0 (0.0-Inf) | 0.6 (0.3-1.3) | 0.8 (0.4-1.9) | 32 (9.1)/18.7 (4.5) | 52/33 |
| >= 105 | 68257 | 2698/34/1201 | To 90-104 | 21257 | 1820/12/399 | 2.2 (2.1-2.3) | 0.9 (0.8-0.9) | 1.1 (0.6-2.1) | 1.1 (0.5-2.1) | 1.1 (1.0-1.2) | 0.8 (0.7-0.9) | 32 (9.1)/37.1 (15.3) | 52/49 |
| 90-104 | 49668 | 4619/28/1142 | To 60-89 | 29642 | 5403/8/957 | 2.3 (2.2-2.4) | 0.8 (0.7-0.8) | 0.5 (0.2-1.0) | 0.6 (0.3-1.3) | 1.6 (1.4-1.7) | 1.0 (0.9-1.1) | 48.9 (10)/58.7 (15.3) | 43/49 |
| 60-89 | 84746 | 15159/105/3244 | To 45-59 | 7084 | 2035/4/349 | 1.9 (1.8-2.0) | 1.0 (0.9-1.0) | 0.4 (0.2-1.2) | 1.0 (0.4-2.6) | 1.4 (1.3-1.6) | 1.2 (1.1-1.3) | 62.2 (13.7)/74.8 (10.2) | 46/54 |
| 45-59 | 12069 | 3915/72/1038 | To 30-44 | 2153 | 836/13/150 | 1.7 (1.6-1.8) | 1.2 (1.1-1.3) | 1.1 (0.6-1.9) | 1.5 (0.8-2.8) | 1.0 (0.9-1.2) | 1.1 (0.9-1.3) | 70.2 (12)/78.7 (9.9) | 46/53 |
| 30-44 | 5948 | 2558/118/746 | To 15-29 | 345 | 164/3/35 | 1.7 (1.5-2.0) | 1.2 (1.0-1.5) | 0.4 (0.1-1.4) | 0.5 (0.1-1.5) | 1.1 (0.8-1.5) | 1.2 (0.9-1.7) | 72.7 (12.7)/81.6 (11.9) | 46/62 |
| 15-29 | 2310 | 1199/191/436 | To <15 | 14 | 10/1/2 | 3.6 (1.9-6.7) | 2.6 (1.4-4.8) | 0.9 (0.1-6.4) | 1.3 (0.2-9.3) | 1.3 (0.3-5.3) | 1.3 (0.3-5.1) | 71.7 (14.9)/82.6 (19.4) | 46/57 |
| ***LMR*** |  |  |  |  |  | *HRs of reclassified individuals versus the HRs in their original* ***better*** *eGFR categories* | | | | | |  |  |
| >= 105 | 32395 | 2091/21/811 | To 60-89 | 3918 | 35/2/21 | 0.2 (0.1-0.2) | 0.5 (0.4-0.8) | 0.9 (0.2-4.0) | 1.1 (0.2-5.3) | 0.2 (0.2-0.4) | 0.3 (0.2-0.5) | 33.1 (10.8)/21.3 (3.1) | 64/34 |
| >= 105 | 32395 | 2091/21/811 | To 90-104 | 53771 | 2401/23/774 | 0.7 (0.6-0.7) | 0.6 (0.6-0.6) | 0.7 (0.4-1.3) | 0.7 (0.4-1.2) | 0.6 (0.5-0.6) | 0.5 (0.5-0.6) | 33.1 (10.8)/34 (11.2) | 64/45 |
| 90-104 | 20124 | 2968/12/606 | To 60-89 | 59298 | 7055/24/1494 | 0.8 (0.8-0.8) | 0.7 (0.7-0.8) | 0.7 (0.4-1.4) | 0.6 (0.3-1.2) | 0.8 (0.8-0.9) | 0.8 (0.8-0.9) | 54.4 (9.1)/51.9 (14.2) | 56/41 |
| 60-89 | 83235 | 14573/94/3146 | To 45-59 | 8783 | 2629/15/452 | 2.0 (1.9-2.1) | 1.0 (0.9-1.0) | 1.4 (0.8-2.5) | 2.7 (1.6-4.7) | 1.5 (1.4-1.7) | 1.2 (1.1-1.4) | 61.9 (13.6)/74.6 (11.4) | 48/39 |
| 45-59 | 12036 | 3909/54/1003 | To 30-44 | 2255 | 854/32/190 | 1.5 (1.4-1.6) | 1.2 (1.1-1.3) | 3.3 (2.1-5.1) | 2.5 (1.6-4.0) | 1.2 (1.0-1.4) | 1.2 (1.0-1.4) | 70.6 (11.6)/75.4 (14.1) | 49/36 |
| 30-44 | 5225 | 2216/82/615 | To 15-29 | 1118 | 521/45/174 | 1.1 (1.0-1.3) | 1.2 (1.1-1.3) | 2.5 (1.7-3.6) | 2.0 (1.4-2.9) | 1.4 (1.2-1.6) | 1.4 (1.1-1.6) | 73 (12.7)/72.9 (14.5) | 48/42 |
| 15-29 | 2349 | 1208/197/444 | To <15 | 29 | 22/1/3 | 3.2 (2.1-4.9) | 1.8 (1.2-2.7) | 0.4 (0.1-2.8) | 0.8 (0.1-5.8) | 0.8 (0.3-2.5) | 0.8 (0.2-2.3) | 71.1 (15.2)/85 (13.8) | 46/45 |
| **Reclassification to better eGFR categories**  ***EKFC*** | | | | | | *HRs of reclassified individuals versus the HRs in their original* ***worse*** *eGFR categories* | | | | | |  |  |
| 90-104 | 49668 | 4619/28/1142 | >= 105 | 112 | 1/0/1 | 0.1 (0.0-0.7) | 0.4 (0.1-2.6) | 0.0 (0.0-Inf) | 0.0 (0.0-Inf) | 0.4 (0.1-2.8) | 0.8 (0.1-5.9) | 48.9 (10)/39.5 (0.5) | 43/0 |
| 60-89 | 84746 | 15159/105/3244 | To 90-104 | 188 | 8/0/5 | 0.2 (0.1-0.3) | 0.7 (0.4-1.5) | 0.0 (0.0-Inf) | 0.0 (0.0-Inf) | 0.5 (0.2-1.3) | 0.7 (0.3-1.7) | 62.2 (13.7)/41.2 (1.4) | 46/0 |
| 45-59 | 12069 | 3915/72/1038 | To 60-89 | 69 | 12/1/5 | 0.3 (0.2-0.6) | 1.0 (0.6-1.7) | 2.1 (0.3-15.1) | 0.4 (0.1-3.2) | 0.6 (0.2-1.4) | 0.4 (0.2-1.1) | 70.2 (12)/43.8 (3.7) | 46/19 |
| 30-44 | 5948 | 2558/118/746 | To 45-59 | 50 | 15/6/8 | 0.3 (0.2-0.6) | 1.1 (0.6-1.8) | 5.5 (2.4-12.5) | 1.1 (0.5-2.5) | 0.7 (0.3-1.4) | 0.6 (0.3-1.1) | 72.7 (12.7)/43.7 (6.6) | 46/34 |
| 15-29 | 2310 | 1199/191/436 | To 30-44 | 54 | 21/6/9 | 0.5 (0.3-0.8) | 1.1 (0.7-1.8) | 1.2 (0.5-2.6) | 0.5 (0.2-1.1) | 0.6 (0.3-1.1) | 0.6 (0.3-1.1) | 71.7 (14.9)/49.3 (8.3) | 46/41 |
| <15 | 1097 | 458/478/251 | To 15-29 | 53 | 20/23/12 | 0.9 (0.6-1.5) | 1.3 (0.8-2.1) | 0.9 (0.6-1.4) | 0.8 (0.5-1.2) | 0.8 (0.5-1.4) | 0.8 (0.5-1.4) | 60.6 (16.1)/53.7 (11.9) | 39/34 |
| ***LMR*** |  |  |  |  |  | *HRs of reclassified individuals versus the HRs in their original* ***worse*** *eGFR categories* | | | | | |  |  |
| <15 | 1092 | 453/476/249 | To 15-29 | 58 | 25/25/14 | 1.0 (0.7-1.6) | 1.5 (1.0-2.3) | 0.9 (0.6-1.3) | 0.7 (0.5-1.1) | 0.9 (0.5-1.5) | 0.9 (0.5-1.5) | 60.7 (16.1)/53.2 (12.2) | 39/38 |

AKI, acute kidney injury; CI, confidence interval; eGFR, estimated glomerular filtration rate; EKFC, European Kidney Function Consortium; F, female; HR, hazard ratio; KF, kidney failure; LMR, Lund-Malmö revised; SCr, serum creatinine in umol/L; *The original eGFR category is based on the CKD-EPIASR-NB 2009 equation; **Age and gender adjusted

**Supplemental Table 5A continued**

| **Non-reclassified** | | | **Reclassified** |  |  | **Outcomes** |  |  | |  |  | | **Patient characteristics, non-reclassified versus reclassified** | |  |  |
| --- | --- | --- | --- | --- | --- | --- | --- | --- | --- | --- | --- | --- | --- | --- | --- | --- |
| **eGFR*** | **n** | **All-cause mortality/KF with KRT/KF/AKI** | **eGFR*** | **n** | **All-cause mortality/KF with KRT/KF/AKI** | **All-cause mortality, HR(95%CI)** | **All-cause mortality, HR(95%CI)**** | | **KF with KRT, HR(95%CI)** | **KF with KRT, HR(95%CI)**** | | **AKI, HR(95%CI)** | **AKI, HR(95%CI)**** | **Age, mean(SD)** | | **F, %** |
| **CKD-EPI_AS_ 2021 equation, reclassification to better eGFR categories***** | | | | | | *HRs of reclassified individuals versus the HRs in their original* ***worse*** *eGFR categories* | | | | | | | |  | |  |
| 90-104 | 61889 | 8480/24/1717 | To >= 105 | 17533 | 1543/12/383 | 0.6 (0.6-0.6) | 1.3 (1.2-1.4) | | 1.8 (0.9-3.6) | 1.5 (0.7-2.9) | | 0.8 (0.7-0.8) | 1.0 (0.9-1.2) | 54.1 (13.5)/46.9 (10.1) | | 46/42 |
| 60-89 | 69042 | 13095/98/2858 | To 90-104 | 22976 | 4107/11/740 | 1.0 (1.0-1.1) | 1.1 (1.1-1.2) | | 0.3 (0.2-0.6) | 0.3 (0.2-0.6) | | 0.8 (0.8-0.9) | 0.8 (0.8-0.9) | 63.4 (14.2)/62.3 (12.9) | | 48/42 |
| 45-59 | 9674 | 3375/79/914 | To 60-89 | 4617 | 1388/7/279 | 0.9 (0.9-1.0) | 0.9 (0.8-0.9) | | 0.2 (0.1-0.4) | 0.2 (0.1-0.5) | | 0.7 (0.6-0.8) | 0.7 (0.6-0.8) | 71.1 (12.6)/71.9 (11.1) | | 47/46 |
| 30-44 | 4665 | 2027/111/622 | To 45-59 | 1678 | 710/16/167 | 1.0 (0.9-1.1) | 0.9 (0.8-1.0) | | 0.4 (0.2-0.7) | 0.5 (0.3-0.9) | | 0.7 (0.6-0.9) | 0.7 (0.6-0.9) | 72.5 (13.4)/74.5 (11.9) | | 47/46 |
| 15-29 | 1898 | 984/177/379 | To 30-44 | 480 | 246/21/68 | 0.9 (0.8-1.1) | 0.8 (0.7-0.9) | | 0.5 (0.3-0.7) | 0.5 (0.3-0.8) | | 0.6 (0.5-0.8) | 0.6 (0.5-0.8) | 70.8 (15.5)/73 (13.8) | | 46/46 |
| <15 | 1054 | 433/479/244 | To 15-29 | 96 | 45/22/19 | 1.9 (1.4-2.6) | 1.2 (0.8-1.6) | | 0.5 (0.3-0.7) | 0.5 (0.4-0.8) | | 0.9 (0.6-1.5) | 0.9 (0.6-1.5) | 59.5 (15.9)/68.6 (15.4) | | 39/38 |

AKI, acute kidney injury; CI, confidence interval; eGFR, estimated glomerular filtration rate; F, female; HR, hazard ratio; KF, kidney failure; SCr, serum creatinine in umol/L;
*The original eGFR category is based on the CKD-EPI_ASR-NB_ 2009 equation; **Age and gender adjusted

**Supplemental Table 5B.** Hazard ratios in reclassified individuals, compared to non-reclassified individuals in the new eGFR category

| **Non-reclassified** | | | **Reclassified** | | | **Outcomes** |  |  |  |  |  | **Patient characteristics, non-reclassified/reclassified** | |
| --- | --- | --- | --- | --- | --- | --- | --- | --- | --- | --- | --- | --- | --- |
| **eGFR*** | **n** | **All-cause mortality/KF with KRT/KF/AKI** | **eGFR*** | **n** | **All-cause mortality/KF with KRT/KF/AKI** | **All-cause mortality, HR(95%CI)** | **All-cause mortality, HR(95%CI)**** | **KF with KRT, HR(95%CI)** | **KF with KRT, HR(95%CI)**** | **AKI, HR(95%CI)** | **AKI, HR(95%CI)**** | **Age, mean(SD)** | **Gender, F %** |
| **Reclassification to worse eGFR categories**  ***EKFC*** | | | | | | *HRs of reclassified individuals versus the HRs in their new (worse) eGFR categories* | | | | | |  |  |
| 60-89 | 84746 | 15159/105/3244 | from >= 105 to 60-89 | 570 | 9/0/6 | 0.1 (0.1-0.2) | 2.4 (1.3-4.7) | 0.0 (0.0-Inf) | 0.0 (0.0-Inf) | 0.3 (0.1-0.7) | 0.6 (0.3-1.4) | 62.2 (13.7)/18.7 (4.5) | 46/33 |
| 90-104 | 49668 | 4619/28/1142 | from >= 105 to 90-104 | 21257 | 1820/12/399 | 1.1 (1.0-1.1) | 1.7 (1.6-1.8) | 1.0 (0.5-2.1) | 0.8 (0.4-1.7) | 0.9 (0.8-1.0) | 1.3 (1.2-1.5) | 48.9 (10)/37.1 (15.3) | 43/49 |
| 60-89 | 84746 | 15159/105/3244 | from 90-104 to 60-89 | 29642 | 5403/8/957 | 1.2 (1.1-1.2) | 1.4 (1.4-1.5) | 0.2 (0.1-0.5) | 0.2 (0.1-0.3) | 0.9 (0.9-1.0) | 1.0 (0.9-1.1) | 62.2 (13.7)/58.7 (15.3) | 46/49 |
| 45-59 | 12069 | 3915/72/1038 | from 60-89 to 45-59 | 7084 | 2035/4/349 | 1.1 (1.1-1.2) | 0.9 (0.9-1.0) | 0.1 (0.0-0.3) | 0.1 (0.0-0.3) | 0.7 (0.6-0.7) | 0.7 (0.6-0.8) | 70.2 (12)/74.8 (10.2) | 46/54 |
| 30-44 | 5948 | 2558/118/746 | from 45-59 to 30-44 | 2153 | 836/13/150 | 1.1 (1.1-1.2) | 0.9 (0.8-1.0) | 0.3 (0.2-0.6) | 0.5 (0.3-0.8) | 0.7 (0.5-0.8) | 0.7 (0.6-0.8) | 72.7 (12.7)/78.7 (9.9) | 46/53 |
| 15-29 | 2310 | 1199/191/436 | from 30-44 to 15-29 | 345 | 164/3/35 | 1.4 (1.2-1.6) | 0.8 (0.7-1.0) | 0.1 (0.0-0.3) | 0.2 (0.1-0.5) | 0.7 (0.5-0.9) | 0.7 (0.5-1.0) | 71.7 (14.9)/81.6 (11.9) | 46/62 |
| <15 | 1097 | 458/478/251 | from 15-29 to <15 | 14 | 10/1/2 | 4.8 (2.6-9.1) | 1.9 (1.0-3.6) | 0.1 (0.0-0.9) | 0.2 (0.0-1.4) | 0.8 (0.2-3.1) | 0.7 (0.2-2.9) | 60.6 (16.1)/82.6 (19.4) | 39/57 |
| ***LMR*** |  |  |  |  |  | *HRs of reclassified individuals versus the HRs in their new (worse) eGFR categories* | | | | | |  |  |
| 60-89 | 83235 | 14573/94/3146 | from >= 105 to 60-89 | 3918 | 35/2/21 | 0.1 (0.0-0.1) | 1.3 (0.9-1.8) | 0.6 (0.1-2.3) | 0.1 (0.0-0.2) | 0.2 (0.1-0.3) | 0.4 (0.2-0.6) | 61.9 (13.6)/21.3 (3.1) | 48/34 |
| 90-104 | 20124 | 2968/12/606 | from >= 105 to 90-104 | 53771 | 2401/23/774 | 0.3 (0.3-0.3) | 1.5 (1.4-1.6) | 0.8 (0.4-1.6) | 0.4 (0.2-1.2) | 0.5 (0.5-0.6) | 1.1 (1.0-1.3) | 54.4 (9.1)/34 (11.2) | 56/45 |
| 60-89 | 83235 | 14573/94/3146 | from 90-104 to 60-89 | 59298 | 7055/24/1494 | 0.7 (0.7-0.7) | 1.3 (1.3-1.4) | 0.4 (0.2-0.6) | 0.2 (0.1-0.3) | 0.7 (0.6-0.7) | 0.9 (0.8-0.9) | 61.9 (13.6)/51.9 (14.2) | 48/41 |
| 45-59 | 12036 | 3909/54/1003 | from 60-89 to 45-59 | 8783 | 2629/15/452 | 1.1 (1.1-1.2) | 0.9 (0.9-1.0) | 0.4 (0.2-0.7) | 0.4 (0.2-0.7) | 0.7 (0.6-0.8) | 0.7 (0.6-0.8) | 70.6 (11.6)/74.6 (11.4) | 49/39 |
| 30-44 | 5225 | 2216/82/615 | from 45-59 to 30-44 | 2255 | 854/32/190 | 1.0 (1.0-1.1) | 0.9 (0.8-1.0) | 1.0 (0.6-1.4) | 0.7 (0.5-1.1) | 0.8 (0.7-0.9) | 0.8 (0.7-0.9) | 73 (12.7)/75.4 (14.1) | 48/36 |
| 15-29 | 2349 | 1208/197/444 | from 30-44 to 15-29 | 1118 | 521/45/174 | 0.9 (0.8-1) | 0.9 (0.8-1.0) | 0.5 (0.3-0.6) | 0.5 (0.4-0.7) | 0.8 (0.7-1.0) | 0.8 (0.7-1.0) | 71.1 (15.2)/72.9 (14.5) | 46/42 |
| <15 | 1092 | 453/476/249 | from 15-29 to <15 | 29 | 22/1/3 | 4.2 (2.7-6.5) | 1.2 (0.8-1.9) | 0.1 (0.0-0.4) | 0.1 (0.0-0.7) | 0.5 (0.1-1.5) | 0.4 (0.1-1.4) | 60.7 (16.1)/85 (13.8) | 39/45 |
| **Reclassification to better eGFR categories**  ***EKFC*** | | | | | | *HRs of reclassified individuals versus the HRs in their new (better) eGFR categories* | | | | | |  |  |
| >= 105 | 68257 | 2698/34/1201 | from 90-104 to >= 105 | 112 | 1/0/1 | 0.2 (0.0-1.4) | 0.1 (0.0-1.0) | 0.0 (0.0-Inf) | 0.0 (0.0-Inf) | 0.5 (0.1-3.3) | 0.4 (0.1-2.8) | 32 (9.1)/39.5 (0.5) | 52/0 |
| 90-104 | 49668 | 4619/28/1142 | from 60-89 to 90-104 | 188 | 8/0/5 | 0.3 (0.2-0.7) | 1.0 (0.5-2.1) | 0.0 (0.0-Inf) | 0.0 (0.0-Inf) | 0.9 (0.4-2.2) | 1.7 (0.7-4.0) | 48.9 (10)/41.2 (1.4) | 43/0 |
| 60-89 | 84746 | 15159/105/3244 | from 45-59 to 60-89 | 69 | 12/1/5 | 0.6 (0.3-1.0) | 2.2 (1.3-3.9) | 9.0 (1.3-64.8) | 3.7 (0.5-26.9) | 1.2 (0.5-3.0) | 1.6 (0.7-3.9) | 62.2 (13.7)/43.8 (3.7) | 46/19 |
| 45-59 | 12069 | 3915/72/1038 | from 30-44 to 45-59 | 50 | 15/6/8 | 0.5 (0.3-0.8) | 1.3 (0.8-2.2) | 17.8 (7.8-41) | 3.6 (1.5-8.3) | 1.1 (0.5-2.2) | 0.9 (0.4-1.8) | 70.2 (12)/43.7 (6.6) | 46/34 |
| 30-44 | 5948 | 2558/118/746 | from 15-29 to 30-44 | 54 | 21/6/9 | 0.6 (0.4-0.9) | 1.3 (0.9-2.1) | 4.9 (2.1-11.0) | 1.4 (0.6-3.1) | 0.9 (0.5-1.8) | 0.8 (0.4-1.6) | 72.7 (12.7)/49.3 (8.3) | 46/41 |
| 15-29 | 2310 | 1199/191/436 | from <15 to 15-29 | 53 | 20/23/12 | 0.7 (0.4-1.1) | 1.4 (0.9-2.2) | 7.5 (4.8-11.5) | 3.6 (2.3-5.6) | 1.3 (0.7-2.3) | 1.3 (0.7-2.4) | 71.7 (14.9)/53.7 (11.9) | 46/34 |
| ***LMR*** |  |  |  |  |  | *HRs of reclassified individuals versus the HRs in their new (better) eGFR categories* | | | | | |  |  |
| 15-29 | 2349 | 1208/197/444 | from <15 to 15-29 | 58 | 25/25/14 | 0.7 (0.5-1.1) | 1.5 (1-2.3) | 6.9 (4.6-10.5) | 3.5 (2.3-5.3) | 1.4 (0.8-2.4) | 1.5 (0.9-2.5) | 71.1 (15.2)/53.2 (12.2) | 46/38 |

AKI, acute kidney injury; CI, confidence interval; eGFR, estimated glomerular filtration rate; EKFC, European Kidney Function Consortium; F, female; HR, hazard ratio; KF, kidney failure; LMR, Lund-Malmö revised; SCr, serum creatinine in umol/L; *The original eGFR category is based on the CKD-EPIASR-NB 2009 equation; **Age and gender adjusted

**Supplemental Table 5B Continued**

| **Non-reclassified** | | | **Reclassified** |  |  | **Outcomes** |  |  |  |  |  | **Patient characteristics, non-reclassified versus reclassified** | | |
| --- | --- | --- | --- | --- | --- | --- | --- | --- | --- | --- | --- | --- | --- | --- |
| **eGFR*** | **n** | **All-cause mortality/KF with KRT/KF/AKI** | **eGFR*** | **n** | **All-cause mortality/KF with KRT/KF/AKI** | **All-cause mortality, HR(95%CI)** | **All-cause mortality, HR(95%CI)**** | **KF with KRT, HR(95%CI)** | **KF with KRT, HR(95%CI)**** | **AKI, HR(95%CI)** | **AKI, HR(95%CI)**** | **Age, mean(SD)** | **F, %** |  |
| **CKD-EPI_AS_ 2021 equation, reclassification to better eGFR categories** | | | | | | *HRs of reclassified individuals versus the HRs in their new (better) eGFR categories* | | | | | |  |  |  |
| >= 105 | 90084 | 4527/46/1606 | from 90-104 to >= 105 | 17533 | 1543/12/383 | 1.6 (1.5-1.7) | 0.6 (0.6-0.7) | 1.3 (0.7-2.4) | 1.3 (0.7-2.7) | 1.1 (1.0-1.2) | 0.7 (0.6-0.8) | 33.1 (11.1)/46.9 (10.1) | 51/42 |  |
| 90-104 | 61889 | 8480/24/1717 | from 60-89 to 90-104 | 22976 | 4107/11/740 | 1.3 (1.3-1.4) | 0.7 (0.7-0.8) | 1.2 (0.6-2.4) | 1.8 (0.9-3.8) | 1.2 (1.1-1.3) | 0.9 (0.8-1.0) | 54.1 (13.5)/62.3 (12.9) | 46/42 |  |
| 60-89 | 69042 | 13095/98/2858 | from 45-59 to 60-89 | 4617 | 1388/7/279 | 1.6 (1.5-1.7) | 1.1 (1.0-1.1) | 1.0 (0.5-2.1) | 1.9 (0.9-4.1) | 1.5 (1.3-1.7) | 1.3 (1.2-1.5) | 63.4 (14.2)/71.9 (11.1) | 48/46 |  |
| 45-59 | 9674 | 3375/79/914 | from 30-44 to 45-59 | 1678 | 710/16/167 | 1.3 (1.2-1.4) | 1.2 (1.1-1.3) | 1.1 (0.7-2) | 1.5 (0.9-2.5) | 1.1 (0.9-1.3) | 1.1 (0.9-1.3) | 71.1 (12.6)/74.5 (11.9) | 47/46 |  |
| 30-44 | 4665 | 2027/111/622 | from 15-29 to 30-44 | 480 | 246/21/68 | 1.1 (1.0-1.3) | 1.1 (1.0-1.3) | 1.8 (1.1-2.8) | 1.8 (1.1-2.9) | 1.0 (0.8-1.3) | 1.0 (0.8-1.3) | 72.5 (13.4)/73.0 (13.8) | 47/46 |  |
| 15-29 | 1898 | 984/177/379 | from <15 to 15-29 | 96 | 45/22/19 | 1.3 (0.9-1.7) | 1.3 (1.0-1.8) | 3.2 (2.1-5.0) | 3.2 (2.0-4.9) | 1.4 (0.9-2.2) | 1.4 (0.9-2.2) | 70.8 (15.5)/68.6 (15.4) | 46/38 |  |

AKI, acute kidney injury; CI, confidence interval; eGFR, estimated glomerular filtration rate; F, female; HR, hazard ratio; KF, kidney failure; SCr, serum creatinine in umol/L;
*The original eGFR category is based on the CKD-EPI_ASR-NB_ 2009 equation; **Age and gender adjusted

**Supplemental Table 6.** Discrimination of eGFR category classification by the different eGFR equations for 10-year outcomes

| **eGFR equation** | **All-cause mortality** | **KF with KRT** | **AKI (all stages)** | **AKI stage 2 or 3** |
| --- | --- | --- | --- | --- |
| CKD-EPI_ASR-NB_ 2009 | 0.570 | 0.897 | 0.599 | 0.548 |
| CKD-EPI_AS_ 2021 | 0.562 | 0.897 | 0.593 | 0.542 |
| EKFC | 0.584 | 0.895 | 0.606 | 0.551 |
| LMR | 0.588 | 0.900 | 0.609 | 0.553 |

All outcomes reflect the Harell’s Concordance index (C-index); AKI, acute kidney injury; CKD-EPI, Chronic Kidney Disease Epidemiology Collaboration; eGFR, Estimated glomerular filtration rate; EKFC, European Kidney Function Consortium; KF, kidney failure; KRT, kidney replacement therapy; LMR, Lund-Malmö revised

**Supplemental Table 7.** Net Reclassification Improvement of the reclassification between all eGFR categories over a follow-up period of maximum 10 years, compared to the classification by the CKD-EPI_ASR-NB_ 2009 equation

| **eGFR equation** | **All-cause mortality** | **KF with KRT** | **AKI (all stages)** | **AKI stage 2 or 3** |
| --- | --- | --- | --- | --- |
| CKD-EPI_AS_ 2021 | -3.9% (-6.1%/2.1%) | -4% (-6.1%/2.1%) | -3.5% (-5.5%/2%) | -2.3% (-4.4%/2.1%) |
| EKFC | 5.5% (7.5%/-2.0%) | -3.4% (-1.4%/-2.0%) | 3.3% (5.2%/-1.9%) | 1.8% (3.8%/-2.0%) |
| LMR | 7.3% (10.1%/-2.8%) | 3.6% (6.4%/-2.8%) | 5.3% (8.0%/-2.7%) | 2.3% (5.1%/-2.8%) |

Outcomes shown as NRI in percentages(net proportion of correctly reclassified individuals with the event to worse CKD stages (NRI+)/ net proportion of correctly reclassified individuals without the event to better CKD stages (NRI-)). Reclassification across all CKD stages were compared. Individuals without follow-up were excluded. Inverse probability weighting (IPW) was used to adjust for the presence of censored data, meaning that individuals without the event with longer follow-up got more weight in the analysis, compared to individuals without the event with shorter follow-up.

AKI, acute kidney injury; CKD-EPI, Chronic Kidney Disease Epidemiology Collaboration; eGFR, estimated glomerular filtration rate; EKFC, European Kidney Function Consortium; NRI, Net Reclassification Improvement; KF, kidney failure; KRT, kidney replacement therapy; LMR, Lund-Malmö revised.

***Sub-analysis in individuals with a measured urinary albumin-creatinine ratio***

**Supplemental Table 8.** Characteristics of individuals with a measured urinary albumin-creatinine ratio

|  |  | **KDIGO Risk classification** | | | | |
| --- | --- | --- | --- | --- | --- | --- |
|  | **Total** | **Low risk** | **Moderate risk** | **High risk** | **Very high risk 1** | **Very high risk 2** |
| **Individuals, n(%)** | 38393 | 27801 (72.4) | 6259 (16.3) | 2264 (5.9) | 1686 (4.4) | 383 (1.0) |
| **Female, n(%)** | 18742 (48.8) | 13753 (49.5) | 3112 (49.7) | 1023 (45.2) | 698 (41.4) | 156 (40.7) |
| **n SCr measurements per individual, median(IQR)** | 2.0 (1.0 to 4.0) | 2.0 (1.0 to 3.0) | 2.0 (1.0 to 5.0) | 3.0 (1.0 to 9.0) | 5.0 (2.0 to 12.0) | 5.0 (2.0 to 11.0) |
| **FU time in years, median(IQR)** | 2.1 (0.1 to 7.0) | 1.6 (0.0 to 6.5) | 3.1 (0.4 to 7.9) | 3.5 (0.9 to 8.4) | 4.0 (1.3 to 8.7) | 3.4 (1.0 to 8.3) |
| **FU time in years (excl. no FU), median(IQR)** | 5.0 (1.0 to 10.5) | 4.3 (0.6 to 10.0) | 6.1 (1.7 to 11.4) | 6.8 (2.5 to 12.1) | 7.4 (3.4 to 12.4) | 7.0 (3.1 to 12.6) |
| 0y, n(%) | 3825 (10.0) | 3261 (11.7) | 440 (7.0) | 83 (3.7) | 34 (2.0) | 7 (1.8) |
| >0-1y, n(%) | 11552 (30.1) | 9009 (32.4) | 1614 (25.8) | 529 (23.4) | 314 (18.6) | 86 (22.5) |
| ≥1y-5y,n(%) | 10239 (26.7) | 6982 (25.1) | 1777 (28.4) | 747 (33.0) | 600 (35.6) | 133 (34.7) |
| ≥5-10y, n(%) | 6925 (18.0) | 4650 (16.7) | 1332 (21.3) | 472 (20.8) | 394 (23.4) | 77 (20.1) |
| ≥10y,n(%) | 5852 (15.2) | 3899 (14.0) | 1096 (17.5) | 433 (19.1) | 344 (20.4) | 80 (20.9) |
| **Age, mean(SD)** | 51.1 (16.7) | 48.6 (15.9) | 54.9 (17.1) | 59.6 (16.8) | 63.8 (15.7) | 60.4 (16.8) |
| 18-30y, n(%) | 5039 (13.1) | 4120 (14.8) | 686 (11.0) | 149 (6.6) | 64 (3.8) | 20 (5.2) |
| 30-50y, n(%) | 11634 (30.3) | 9471 (34.1) | 1446 (23.1) | 408 (18.0) | 230 (13.6) | 79 (20.6) |
| 50-65y, n(%) | 12466 (32.5) | 9243 (33.2) | 1998 (31.9) | 677 (29.9) | 443 (26.3) | 105 (27.4) |
| ≥65y, n(%) | 9254 (24.1) | 4967 (17.9) | 2129 (34.0) | 1030 (45.5) | 949 (56.3) | 179 (46.7) |
| BMI^a^, median(IQR) | 26.0 (24.0 to 29.0) | 26.0 (24.0 to 29.0) | 26.0 (23.0 to 29.0) | 26.0 (24.0 to 29.0) | 26.0 (24.0 to 30.0) | 25.0 (22.0 to 27.5) |
| HbA1c %^b^, median(IQR) | 5.8 (5.4 to 6.6) | 5.7 (5.4 to 6.5) | 6.0 (5.5 to 6.9) | 6.0 (5.6 to 6.8) | 5.9 (5.6 to 6.7) | 5.7 (5.4 to 6.3) |
| HbA1c mmol/mol^c^, median(IQR) | 38.0 (34.0 to 43.0) | 37.0 (34.0 to 42.0) | 40.0 (35.0 to 49.0) | 40.0 (36.0 to 49.0) | 40.0 (36.0 to 49.0) | 38.0 (34.0 to 45.0) |
| LDL mmol/L^d^, median(IQR) | 2.7 (2.1 to 3.5) | 2.8 (2.1 to 3.5) | 2.6 (2.0 to 3.3) | 2.5 (1.9 to 3.3) | 2.4 (1.8 to 3.2) | 2.2 (1.6 to 3.0) |
| UACR mg/mmol, median(IQR) | 0.9 (0.5 to 2.3) | 0.7 (0.4 to 1.2) | 4.3 (2.5 to 8.3) | 9.1 (2.2 to 42.1) | 11.0 (4.4 to 32.4) | 61.0 (34.2 to 105.5) |
| SCr umol/L, median(IQR) | 75.0 (62.0 to 90.0) | 71.0 (60.0 to 83.0) | 81.0 (63.0 to 99.0) | 111.0 (85.0 to 132.0) | 158.0 (136.0 to 193.0) | 287.0 (224.0 to 407.5) |
| **eGFR** |  |  |  |  |  |  |
| CKD-EPI_ASR-NB_ 2009 equation | 90.1 (27.7) | 97.8 (20.3) | 84.6 (29.0) | 63.6 (30.3) | 35.2 (9.9) | 17.6 (7.1) |
| CKD-EPI_AS_ 2021 equation | 93.1 (26.5) | 101.0 (18.6) | 87.5 (26.5) | 66.2 (28.4) | 37.4 (10.4) | 18.8 (7.5) |
| EKFC equation | 84.8 (24.9) | 92.0 (17.9) | 79.3 (25.4) | 60.2 (26.8) | 34.4 (9.4) | 18.1 (6.9) |
| LMR equation | 80.7 (23.9) | 87.4 (17.7) | 76.3 (23.7) | 57.9 (25.3) | 32.1 (9.5) | 17.3 (5.6) |
| **Outcomes** |  |  |  |  |  |  |
| All-cause mortality, n(%) | 4985 (13.0) | 2170 (7.8) | 1176 (18.8) | 692 (30.6) | 761 (45.1) | 186 (48.6) |
| KFRT, n(%) | 427 (1.1) | 26 (0.1) | 42 (0.7) | 65 (2.9) | 167 (9.9) | 127 (33.2) |
| AKI (any stage), n(%) | 944 (2.5) | 330 (1.2) | 206 (3.3) | 135 (6.0) | 213 (12.6) | 60 (15.7) |

UACR, Urinary albumin-to-creatinine ratio; AKI, acute kidney injury; BMI, Body mass index; CKD-EPI, Chronic Kidney Disease Epidemiology Collaboration; FU, follow-up; HbA1c, Glycated hemoglobin; SCr, Serum creatinine; eGFR, Estimated glomerular filtration rate; EKFC, European Kidney Function Consortium; KFRT, kidney failure with kidney replacement therapy; KTx, kidney transplantation; LDL, Low-Density Lipoprotein; LMR, Lund-Malmö revised; Percentage missing: a) 89.6%, b) 80.5%, c) 34.6%, d) 21.7%

**Supplemental Table 9.** Differences in KDIGO CKD risk category classification between the CKD-EPI_ASR-NB_ 2009, and the A) CKD-EPI_AS_ 2021 equation, B) EKFC equation, C) LMR equation

A)

|  |  | **CKD-EPI_ASR-NB_ 2009 equation** | | | | | |  | |
| --- | --- | --- | --- | --- | --- | --- | --- | --- | --- |
|  |  | **Low risk** | **Moderate risk** | **High risk** | **Very high risk 1** | **Very high risk 2** | **Total, n(%)** | |  |
| **CKD-EPI_AS_ 2021 equation** | **Low risk** | 27806 (100.0) | 534 (8.5) |  |  |  | 28340 (73.8) | |  |
|  | **Moderate risk** |  | 5733 (91.5) | 364 (16.0) |  |  | 6097 (15.9) | |  |
|  | **High risk** |  |  | 1904 (84.0) | 231 (13.7) |  | 2135 (5.6) | |  |
|  | **Very high risk 1** |  |  |  | 1458 (86.3) | 35 (9.1) | 1493 (3.9) | |  |
|  | **Very high risk 2** |  |  |  |  | 349 (90.9) | 349 (0.9) | |  |
|  | **Total, n(%)** | 27806 (72.4) | 6267 (16.3) | 2268 (5.9) | 1689 (4.4) | 384 (1.0) | 38414 | |  |

B)

|  |  | **CKD-EPI_ASR-NB_ 2009 equation** | | | | |  |
| --- | --- | --- | --- | --- | --- | --- | --- |
|  |  | **Low risk** | **Moderate risk** | **High risk** | **Very high risk 1** | **Very high risk 2** | **Total, n(%)** |
| **EKFC equation** | **Low risk** | 27207 (97.8) | 14 (0.2) |  |  |  | 27221 (70.9) |
|  | **Moderate risk** | 599 (2.2) | 5876 (93.8) | 16 (0.7) |  |  | 6491 (16.9) |
|  | **High risk** |  | 377 (6.0) | 2092 (92.2) | 25 (1.5) |  | 2494 (6.5) |
|  | **Very high risk 1** |  |  | 160 (7.1) | 1644 (97.3) | 12 (3.1) | 1816 (4.7) |
|  | **Very high risk 2** |  |  |  | 20 (1.2) | 372 (96.9) | 392 (1.0) |
|  | **Total, n(%)** | 27806 (72.4) | 6267 (16.3) | 2268 (5.9) | 1689 (4.4) | 384 (1.0) | 38414 |

C)

|  |  | **CKD-EPI_ASR-NB_ 2009 equation** | | | | |  |
| --- | --- | --- | --- | --- | --- | --- | --- |
|  |  | **Low risk** | **Moderate risk** | **High risk** | **Very high risk 1** | **Very high risk 2** | **Total, n(%)** |
| **LMR equation** | **Low risk** | 27040 (97.2) |  |  |  |  | 27040 (70.4) |
|  | **Moderate risk** | 766 (2.8) | 5785 (92.3) |  |  |  | 6551 (17.1) |
|  | **High risk** |  | 482 (7.7) | 1957 (86.3) |  |  | 2439 (6.3) |
|  | **Very high risk 1** |  |  | 311 (13.7) | 1619 (95.9) | 6 (1.6) | 1936 (5.0) |
|  | **Very high risk 2** |  |  |  | 70 (4.1) | 378 (98.4) | 448 (1.2) |
|  | **Total, n(%)** | 27806 (72.4) | 6267 (16.3) | 2268 (5.9) | 1689 (4.4) | 384 (1.0) | 38414 |

Green and orange represent individuals classified as having better or worse KDIGO risk classification compared to the classification according to the CKD-EPI_ASR-NB_ 2009 equation, respectively.

CKD-EPI, Chronic Kidney Disease Epidemiology Collaboration; EKFC, European Kidney Function Consortium; LMR, Lund-Malmö revised

**Supplemental Table 10.** Discrimination of KDIGO CKD risk category by the different eGFR equations for 10-year outcomes

| **eGFR equation** | **All-cause mortality** | **KF with KRT** | **AKI (all stages)** |
| --- | --- | --- | --- |
| CKD-EPI_ASR-NB_ 2009 | 0.668 | 0.910 | 0.681 |
| CKD-EPI_AS_ 2021 | 0.664 | 0.913 | 0.674 |
| EKFC | 0.677 | 0.907 | 0.680 |
| LMR | 0.679 | 0.910 | 0.683 |

All outcomes reflect the Harell’s Concordance index (C-index); AKI, acute kidney injury; CKD-EPI, Chronic Kidney Disease Epidemiology Collaboration; eGFR, Estimated glomerular filtration rate; EKFC, European Kidney Function Consortium; KF, kidney failure; KRT, kidney replacement therapy; LMR, Lund-Malmö revised

**Supplemental Table 11.** Net Reclassification Improvement of the reclassification between all KDIGO CKD risk categories over a follow-up period of 10 years, compared to the classification by the CKD-EPI_ASR-NB_ 2009 equation

| **eGFR equation** | **All-cause mortality** | **KF with KRT** | **AKI (all stages)** |
| --- | --- | --- | --- |
| CKD-EPI_AS_ 2021 | -4.0% (-8.0%/4.0%) | -0.8% (-4.7%/3.9%) | -7.8% (-11.7%/3.9%) |
| EKFC | 5.0% (7.4%/-2.5%) | -3.6% (-0.9%/-2.6%) | 0.2% (2.7%/-2.5%) |
| LMR | 6.7% (11.3%/-4.6%) | 2.8% (7.3%/-4.5%) | 3.0% (7.5%/-4.5%) |

Outcomes shown as NRI in percentages(net proportion of correctly reclassified individuals with the event to worse CKD stages (NRI+)/ net proportion of correctly reclassified individuals without the event to better CKD stages (NRI-)). Reclassification across all CKD stages were compared. Individuals without follow-up were excluded. Inverse probability weighting (IPW) was used to adjust for the presence of censored data, meaning that individuals without the event with longer follow-up got more weight in the analysis, compared to individuals without the event with shorter follow-up.

AKI, acute kidney injury; CKD-EPI, Chronic Kidney Disease Epidemiology Collaboration; eGFR, estimated glomerular filtration rate; EKFC, European Kidney Function Consortium; NRI, Net Reclassification Improvement; KF, kidney failure; KRT, kidney replacement therapy; LMR, Lund-Malmö revised.

**Supplemental references**

1. Levey AS, Stevens LA, Schmid CH, et al. A new equation to estimate glomerular filtration rate. *Ann Intern Med*. May 5 2009;150(9):604-12. doi:10.7326/0003-4819-150-9-200905050-00006

2. Pottel H, Björk J, Courbebaisse M, et al. Development and Validation of a Modified Full Age Spectrum Creatinine-Based Equation to Estimate Glomerular Filtration Rate : A Cross-sectional Analysis of Pooled Data. *Ann Intern Med*. Feb 2021;174(2):183-191. doi:10.7326/m20-4366

3. Delanaye P, Vidal-Petiot E, Björk J, et al. Performance of creatinine-based equations to estimate glomerular filtration rate in White and Black populations in Europe, Brazil and Africa. *Nephrol Dial Transplant*. Jan 23 2023;38(1):106-118. doi:10.1093/ndt/gfac241

4. Björk J, Grubb A, Sterner G, Nyman U. Revised equations for estimating glomerular filtration rate based on the Lund-Malmö Study cohort. *Scand J Clin Lab Invest*. May 2011;71(3):232-9. doi:10.3109/00365513.2011.557086

5. Inker LA, Eneanya ND, Coresh J, et al. New Creatinine- and Cystatin C-Based Equations to Estimate GFR without Race. *N Engl J Med*. Nov 4 2021;385(19):1737-1749. doi:10.1056/NEJMoa2102953

6. Kidney Disease: Improving Global Outcomes Acute Kidney Injury Work Group. KDIGO Clinical Practice Guideline for Acute Kidney Injury. Kidney Int Suppl 2012; 2: 1–138.

7. Niemantsverdriet M, Khairoun M, El Idrissi A, et al. Ambiguous definitions for baseline serum creatinine affect acute kidney diagnosis at the emergency department. *BMC Nephrol*. Nov 8 2021;22(1):371. doi:10.1186/s12882-021-02581-x

8. Pencina MJ, D'Agostino RB, Sr., Steyerberg EW. Extensions of net reclassification improvement calculations to measure usefulness of new biomarkers. *Stat Med*. Jan 15 2011;30(1):11-21. doi:10.1002/sim.4085
